# Reversing enhancer RNA–mediated *IKBKE* gene repression enables synthetic anticancer immunity in prostate cancer models

**DOI:** 10.1172/JCI190928

**Published:** 2026-01-16

**Authors:** Xiang Li, Rui Sun, Hao Li, Jacob J. Orme, Xu Zhang, Yu Hou, Sean S. Park, Yu Zhang, Yi He, Liguo Wang, Veronica Rodriguez-Bravo, Josep Domingo-Domenech, Shancheng Ren, Dan Xia, Guanghou Fu, Zhankui Jia, Haojie Huang

**Affiliations:** 1 Department of Urology, Institute of Urologic Science and Technology, The First Affiliated Hospital, Zhejiang University School of Medicine, Hangzhou, China.; 2 Department of Urology, the First Affiliated Hospital of Zhengzhou University, Zhengzhou, China.; 3 Department of Biochemistry and Molecular Biology, and; 4 Department of Oncology, Mayo Clinic College of Medicine and Science, Rochester, Minnesota, USA.; 5 Robert and Arlene Kogod Center on Aging, Mayo Clinic, Rochester, Minnesota, USA.; 6 Department of Pulmonology, Children’s Hospital, National Clinical Research Center for Child Health, Liangzhu Laboratory, Zhejiang University School of Medicine, Hangzhou, China.; 7 Department of Radiation Oncology, Mayo Clinic College of Medicine and Science, Rochester, Minnesota, USA.; 8 The First Hospital of Jiaxing, Jiaxing, China.; 9 Division of Biomedical Statistics and Informatics, Mayo Clinic College of Medicine and Science, Rochester, Minnesota, USA.; 10 Department of Urology, Changzheng Hospital, Shanghai, China.

**Keywords:** Immunology, Oncology, Therapeutics, Innate immunity, Prostate cancer

## Abstract

Immunotherapy has been effective in many cancer types but has failed in multiple clinical trials in prostate cancers, with the underlying mechanisms remaining largely unclear. Here, we demonstrate that androgen receptor pathway inhibitor (ARPI) plus irradiation (IR) triggered robust anticancer immunity in prostate cancers in both patients and mice. We show that androgen-activated AR suppressed innate immune signaling by inducing inhibitor of nuclear factor kappa-B kinase subunit epsilon (*IKBKE*) gene repression through HDAC2 interaction with an *IKBKE* enhancer RNA (*IKBKE* eRNA, or *IKBKE-e*). ARPI treatment caused *IKBKE* derepression and enhanced an IR-induced innate immune response via action of RIG-I and MDA5 dsRNA sensors. *IKBKE-e* ablation largely enhanced innate immunity in prostate cancer cells in culture and anticancer immunity in mice. Our results revealed AR, HDAC2, and *IKBKE* eRNA as critical intrinsic immune suppressors in prostate cancer cells, suggesting that rejuvenating inhibitor of nuclear factor kappa-B kinase subunit epsilon (IKKε) signaling by targeting *IKBKE-e* is an actionable strategy to elicit synthetic anticancer immunity in immunologically “cold” cancers such as prostate cancer.

## Abstract 2

Suppression of IKKε and innate immune response

## Introduction

Immunotherapy has revolutionized the treatment of many cancers (1, 2). The blockade of programmed death 1 (PD-1) and programmed death ligand 1/-2 (PD-L1/2) interaction has been the major immunotherapy paradigm with hundreds of approved clinical indications. Additional immune checkpoint inhibitors (ICIs) blocking cell-cell signaling by targeting CTLA4, LAG-3, and others have also shown promise in multiple indications (3–5). Unfortunately, these approaches have failed in prostate cancer clinical trials. Large phase III trials of single-agent pembrolizumab in KEYNOTE-199 (6) and dual-checkpoint blockade with nivolumab and ipilimumab in CheckMate 650 (7) have reported poor response rates. Methods to overcome the limitations of prostate cancer–targeted ICI are needed.

The androgen receptor (AR) is a member of the steroid hormone receptor superfamily that primarily functions as a transcription activator (8). An early study indicates that the AR influences innate and adaptive immune systems by modulating the function of neutrophils and the development of T and B cells in mice (9). Intriguingly, it has been shown previously that the potent synthetic androgen methyltestosterone can increase the immunogenicity in LNCaP prostate cancer cell line via a viral mimicry response (10). A recent study further links AR activity in T cells to the ICI efficacy in metastatic castration-resistant prostate cancer (CRPC) (11). However, the aforementioned immune tolerance propensity in prostate cancer cannot simply be explained by the AR action in T cells. Indeed, the second-generation AR pathway inhibitors (ARPIs) have not improved immunotherapy response rates in the clinic (12–14). Alternatively, cell damage to produce neoantigens and inflammatory responses is often recognized as a mechanism to be harnessed for immunotherapy. Unfortunately, monotherapies including chemotherapy (15, 16), radiotherapy (17, 18), poly (ADP-ribose) polymerase (PARP) inhibition (19), and cellular immune therapy (20–22) have also failed to meaningfully improve ICI responses in prostate cancer.

Despite the failure of ICIs, antitumor immunity could be critical in prostate cancer therapy. The OPeRATIC trial (NCT02816983) showed largely improved outcomes in patients with oligometastatic CRPC treated with metastasis-directed irradiation (IR) and ARPI in patients who experienced increased levels of CD8^+^CD11a^hi^ T cells in the peripheral circulation (23). These T cell populations have been shown to be critical in response to ICIs. ICI therapy targets the adaptive immune system; innate immune signaling is prerequisite for this adaptive immunity (24). The initiation of innate immune signaling is a consequence of cellular stress–induced activation of pathogen-associated molecular pattern (PAMP) receptors such as the TLRs, melanoma differentiation-associated protein (MDA5), retinoic acid-inducible gene (RIG-I), and cyclic GMP-AMP synthase/stimulator of interferon response cGAMP interactor 1 (cGAS/STING) (25, 26). These pathways can initiate IFN signaling via dsRNA (e.g., viral mimicry) to alert nearby innate immune cells such as DCs, macrophages, and NK cells that form the first line of defense against malignant cells and pathogens (27). Thus, ICI resistance may stem from suppression of the innate immune response at the prostate cancer cell level.

Enhancers, largely localized in the intergenic or intronic regions, are a group of major DNA elements that are essential for gene transcription, especially those tissue-specific genes (28). RNA transcribed from enhancers (termed eRNA) has been suggested to activate gene transcription by facilitating the recruitment or activation of transcription factors and coactivators including c-Jun, YY1, AR, CBP/p300, pTEFb, and SWI-SNF complexes, among others (29–34). However, whether eRNA plays roles in transcription repression remains poorly understood. In the present study, we found that an eRNA transcribed from a putative enhancer in the *IKBKE* gene locus was important for AR repression of *IKBKE* transcription and that the effect of *IKBKE*-eRNA was mediated through its facilitation in the recruitment of the transcription repressor HDAC2. We further showed that dual IR/ARPI treatment not only induced an abscopal antitumor effect in mice and immune activation in patients, but also sensitized cancer cells to ICI immunotherapy in a prostate cancer syngeneic mouse model.

## Results

### Dual radiation and antiandrogen therapy influences T cell activation that predicts outcomes in patients with CRPC and in an immunocompetent mouse model.

**Figure 1:**
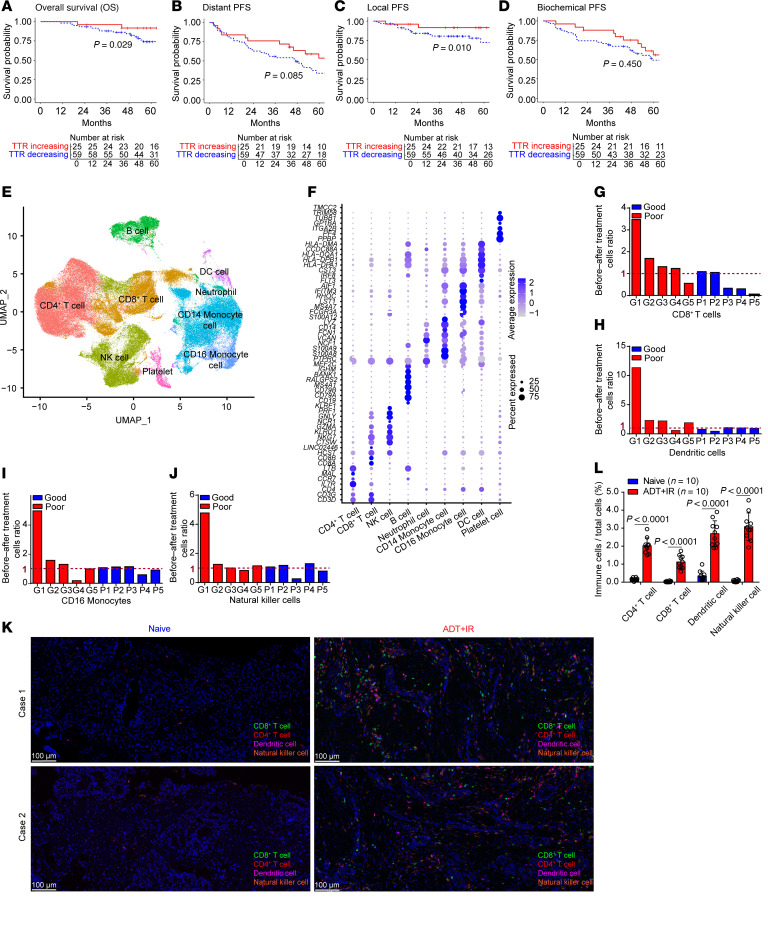
Dual IR and antiandrogen therapy activates the immune response and predicts better outcomes for patients with prostate cancer. (**A**–**D**) Patients’ OS (HR, 0.44 [95% CI: 0.18–1.1]) (**A**), distant PFS (HR, 0.60 [95% CI: 0.34–1.04]) (**B**), local PFS (HR, 0.54 [95% CI: 0.2–1.45]) (**C**), and biochemical PFS (HR, 0.67 [95% CI: 0.36–1.23]) (**D**) were analyzed using the data on 84 patients from the Mayo Clinic who had undergone radiotherapy. Significance in **A**–**D** determined by log-rank test.(**E** and **F**) scRNA-Seq analysis of PBMCs from 10 patients at D0 and D14 after treatment. (**E**) Uniform manifold approximation and projection (UMAP) plot of the cell populations. (**F**) Dot plot showing the expression of canonical markers of each cell type. (**G**–**J**) Cell ratios of CD8^+^ T cells (**G**), DCs (**H**), CD16^+^ monocytes (**I**), and NK cells (**J**) in samples from patients with prostate cancer. (**K** and **L**) mIHC analysis of immune cells infiltrated into prostate cancer patient tissues before and after a 3-month treatment with ADT/IR (**K**), with quantitative analysis (**L**). Scale bars: 100 μm. Data are displayed as the mean ± SEM in **L**. Significance in **L** was determined by 2-tailed *t* test.

We previously observed that patients with oligometastatic CRPC experience an effective treatment response from a combination of ARPIs and IR despite prior resistance to AR-directed therapy (23). We further observed that increasing levels of tumor-reactive CD8^+^CD11a^hi^ T cells (T_TR_) predict improved clinical outcomes. We hypothesized that the patients with increasing T_TR_ after IR would continue to enjoy long-term improvement in overall survival (OS) and local progression-free survival (PFS) in further follow-up. “Local” refers to the absence of recurrence in the primary tumor’s vicinity, showing the treatment’s success in controlling the disease at its starting point. In our 5-year follow-up of these 84 patients (Figure 1, A–D), patients with increasing peripheral blood T_TR_ 2 weeks after IR and ARPI therapy had a superior 5-year OS (91.8% versus 74.4%, *P* = 0.029) and local PFS (91.6% vs. 69.6%, *P* = 0.01). These patients also experienced a trend toward improved 5-year distant PFS (53.6% versus 32.8%, *P* = 0.085). There was no improvement in 5-year biochemical PFS (56% versus 46.7%, *P* = 0.45) in the cohort. Overall, these were excellent long-term outcomes in a cohort of patients with metastatic CRPC.

To better understand the interplay of IR and ARPI in patients experiencing a good response to dual IR and ARPI therapy, we performed single-cell RNA-Seq (scRNA-Seq) analysis of PBMCs from 10 patients at days 0 and 14 (D0 and D14) of treatment, including from 5 patients with a poor response (short responders) and 5 with a good response (long responders) (Figure 1, E and F). In most patients who had a good response, we found that IR plus ARPI treatment was associated with an increase in the number of mononuclear cells, especially CD8^+^ T cells (Figure 1, G–J). We also examined the level of infiltrated mononuclear cells in samples from a cohort of patients with treatment-naive disease versus those treated with androgen deprivation therapy (ADT) and IR for locally advanced prostate cancer. We demonstrated that ADT plus IR treatment substantially increased infiltration of CD4^+^ and CD8^+^ DCs and NK cells in patients’ samples (Figure 1, K and L). Together, these findings indicate that AR pathway inhibition in combination with IR treatment increased CD8^+^ T cells in PBMCs from patients with CRPC and T cell infiltration in prostate cancer patient specimens.

Prostate cancers are often studied in mouse models lacking a functional immune system. To better define the molecular mechanisms that link the contribution of radiotherapy and ARPIs to both systemic immunity and tumor immune microenvironment enrichment, we examined the effects of IR or ARPI alone or in combination in the Myc-CaP syngeneic murine prostate cancer model (Supplemental Figure 1, A–D; supplemental material available online with this article; https://doi.org/10.1172/JCI190928DS1). Given that anticancer T cells were elevated in the peripheral blood in patients who were better responders (Figure 1G), we also determined whether dual ARPI and radiation treatment could trigger the abscopal effect on tumor growth in mice by including a group of tumors without direct IR (right group, Supplemental Figure 1A). In the group of tumors treated directly with IR or in combination with the anti-androgen enzalutamide (ENZ) (left group, Supplemental Figure 1A), we found that while either IR or ENZ treatment alone reduced tumor growth, the inhibition was much more robust in the combination treatment group (Supplemental Figure 1, A–D). We next examined whether CD4^+^ and CD8^+^ T cells were similarly elevated in the peripheral blood of mice treated with IR and ENZ (IR/ENZ). IR or ENZ treatment alone increased CD45^+^CD4^+^ lymphocytes in PBMCs of mice, but combined IR plus ENZ largely increased CD45^+^CD4^+^ lymphocytes in mouse PBMCs (Supplemental Figure 1E). Similar results were obtained for CD45^+^CD8^+^ lymphocytes in mice with tumors treated with IR or ENZ alone or in combination (Supplemental Figure 1F). The observations in the Myc-CaP mouse model recapitulate the findings in patients with prostate cancer.

Anticancer immune responses require T cell infiltration into the tumor microenvironment. Previous studies have shown that the prostate tumor microenvironment is inhospitable for tumor infiltrate and that this may be a key reason for ICI resistance in prostate cancer. We sought to determine whether immune cell infiltration could be improved by combined IR and ARPI therapy. We isolated grafted Myc-CaP tumors after 21 days of treatment, dissociated tissues, and measured the number of CD4^+^ and CD8^+^ T cells in each sample by flow cytometry. We found that infiltrating CD4^+^ and CD8^+^ T cells were rare at baseline but were increased in tumors from mice treated with ENZ alone or with IR alone. Notably, both cell types increased much more robustly with dual IR/ENZ treatment (Supplemental Figure 1, G and H).

In agreement with the increased CD8^+^ T cells in the peripheral blood of patients or mice with CRPC following tumors treated with ARPI plus IR (Figure 1G and Supplemental Figure 1F), we observed obvious abscopal effects of dual treatment on Myc-CaP tumor growth in the mice (Supplemental Figure 1, A–D). Notably, dual treatment also significantly increased both CD45^+^CD4^+^ and CD45^+^CD8^+^ lymphocyte numbers in tumors without direct IR treatment (Supplemental Figure 1, G and H). Thus, dual radiation and antiandrogen induced abscopal immune activation and anticancer activity in mice, consistent with our observations in patients.

### Dual radiation and antiandrogen treatment activates innate immune signaling in prostate cancer cells.

**Figure 2:**
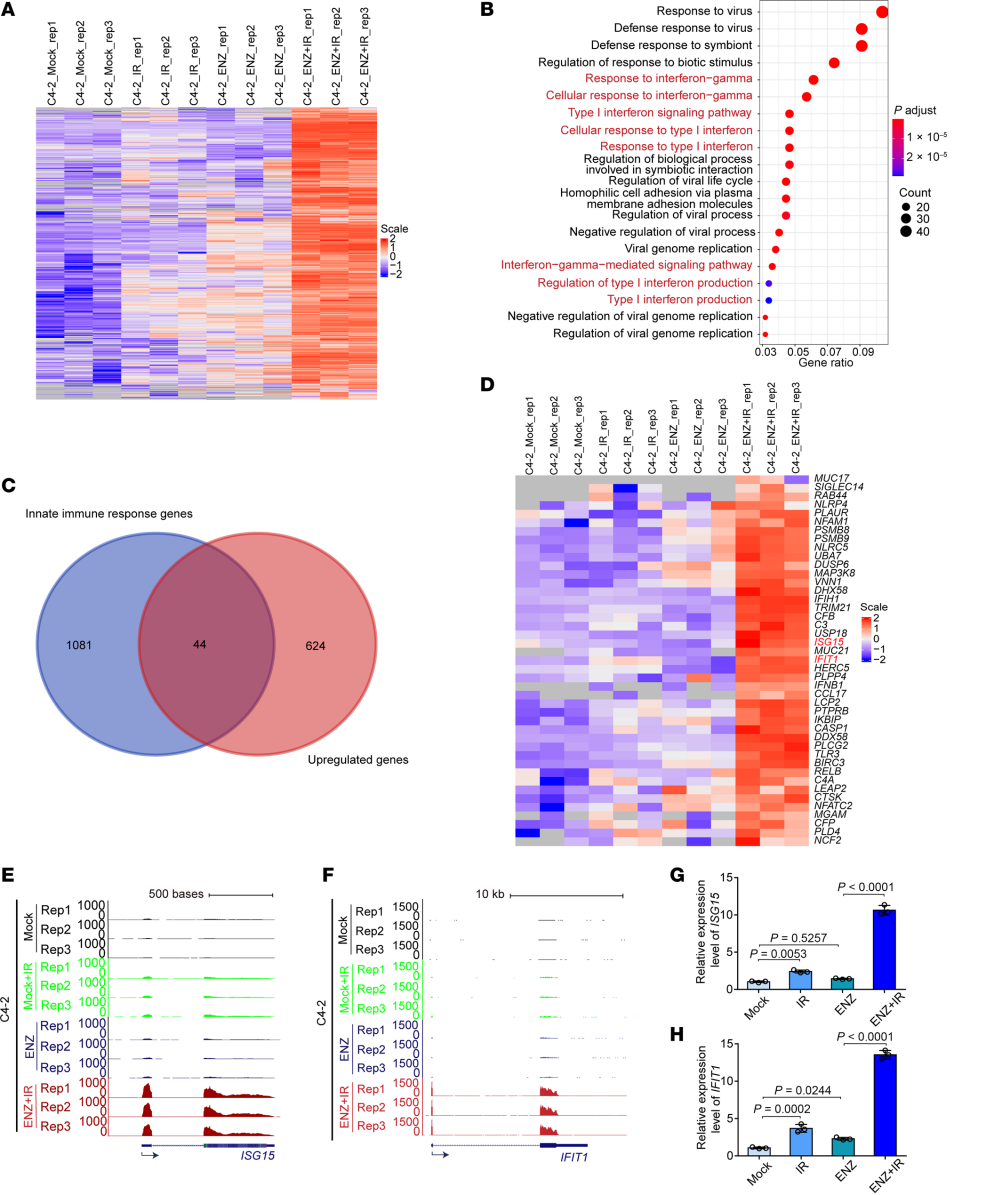
Dual IR and antiandrogen therapy activates prostate cancer innate immune signaling. (**A**) Heatmap showing upregulated genes in C4-2 cells treated with IR (6 Gy), ENZ (10 μM, 48 hours), or a combination of both. (**B**) Bubble plots of the top GO terms of biological processes using the upregulated genes shown in **A**. (**C**) Venn diagram showing the overlap of innate immune genes with upregulated genes by long-term ENZ treatment and radiotherapy in C4-2 cells. (**D**) Heatmap showing the overlapped genes shown in **C**. (**E** and **F**) UCSC Genome Browser screenshot of RNA-Seq tracks for *ISG15* (**E**) and *IFIT1* (**F**) from C4-2 cells treated with IR (6 Gy), ENZ (10 μM, 48 hours), or their combination. (**G** and **H**) RT-qPCR analysis of *ISG15* (**G**) and *IFIT1* (**H**) in C4-2 cells treated with IR (6 Gy), ENZ (10 μM, 48 hours), or their combination. Data are displayed as the mean ± SD of triplicate experiments in **G** and **H**, and was determined by 1-way ANOVA with Tukey’s correction applied for multiple comparisons.

The development of anticancer immunity requires multiple steps, including intrinsic cancer cell signaling, innate immune surveillance, and adaptive immune expansion and activity. We postulated that combined AR suppression and radiation may lead to increased intrinsic cancer cell signaling changes. To determine the effect of combined ARPI and IR on prostate cancer cells, we treated C4-2 prostate cancer cells with vehicle, IR alone, ENZ alone, or combined IR and ENZ and performed RNA-Seq analysis on isolated cells. Among the genes largely upregulated uniquely by IR/ENZ (Figure 2A), we performed Gene Ontology (GO) analysis and found that the combination of IR and ENZ led to significantly increased intrinsic immune signaling relating to PAMP engagement and IFN signaling (Figure 2B). There was a substantial overlap of identified innate immunity–related genes and transcripts upregulated by combined ENZ/IR treatment, including the key type I IFN signaling genes *ISG15* (interferon-stimulated Gene 15) and IFN-induced protein with tetratricopeptide repeats 1 (*IFIT1*) (Figure 2, C–F). Next, we validated the elevation of these transcripts by reverse transcription quantitative PCR (RT-qPCR) (Figure 2, G and H). We further recapitulated these findings in the Myc-CaP murine prostate cancer model with RNA-Seq (Supplemental Figure 2, A–H). Thus, dual treatment of ENZ and IR induced an innate immune response in both human and mouse prostate cancer cells.

### The AR blocks prostate cancer cell IFN signaling through suppression of IKKε.

**Figure 3:**
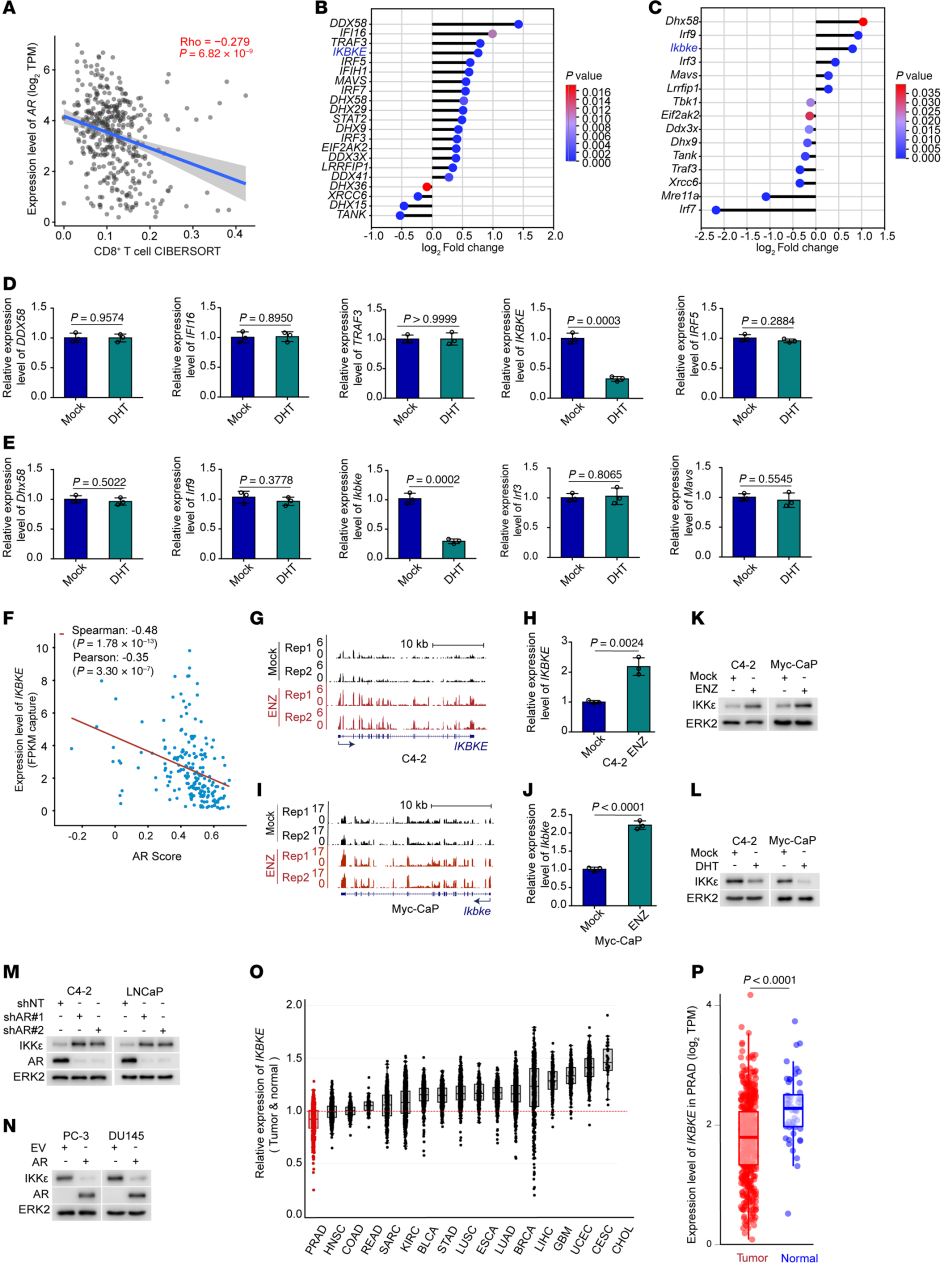
The AR negatively regulates prostate cancer cell IFN signaling via suppression of IKKε expression. (**A**) Scatter plots showing the negative correlation between CD8^+^ T cell infiltration and AR scores in prostate cancer specimens of the TCGA cohort. (**B** and **C**) Gene expression analysis of innate immune genes from C4-2 (**B**) and Myc-CaP (**C**) cells treated with ENZ (10 μM, 48 hours). (**D** and **E**) RT-qPCR analysis of the indicated genes from C4-2 (**D**) and Myc-CaP (**E**) cells cultured in CSS medium supplemented with or without DHT (10 nM, 24 hours). (**F**) Scatter plots showing the negative correlation between the AR score and *IKBKE* mRNA expression from TCGA prostate cancer samples. (**G**–**J**) UCSC Genome Browser screenshot of RNA-Seq tracks from C4-2 (**G**) and Myc-CaP (**I**) cells treated with vehicle or ENZ (10 μM, 48 hours). RT-qPCR analysis of *IKBKE* gene from C4-2 (**H**) and Myc-CaP (**J**) cells treated with vehicle or ENZ (10 μM, 48 hours). (**K**) Western blot analysis of IKKε protein levels in C4-2 and Myc-CaP cells treated with vehicle or ENZ (10 μM) for 48 hours. (**L**) Western blot analysis of IKKε protein levels in C4-2 and Myc-CaP cells cultured in CSS supplemented with vehicle or DHT (10 nM, 24 hours). (**M**) Western blot analysis of IKKε protein levels in C4-2 and LNCaP cells transfected with nonspecific RNA (shNT) or AR-specific shRNAs. (**N**) Western blot analysis of IKKε protein levels in PC-3 and DU145 cells transfected with empty vector or AR plasmids. (**O**) Box plots showing the relative expression of *IKBKE* across various tumor types from TCGA database. (**P**) Dot plots of *IKBKE* gene expression in tumors and normal tissues from TCGA prostate cancer samples. Data are displayed as the mean ± SD of triplicate experiments in **D**, **E**, **H**, and **J** and as the mean ± SEM in **P**. Significance in **D**, **E**, **H**, **J**, and **P** was determined by 2-tailed *t* test.

The above combination of IR/ENZ induced a significant systemic type I IFN response in both humans and mice. Further, IR/ENZ improved the infiltration of adaptive immune cells into the tumor microenvironment in our mouse model. In agreement with the fact that the AR is a major driver of prostate cancer and is the therapeutic target of ENZ, CIBERSORT analysis showed that AR expression levels negatively correlated with CD8^+^ T cell infiltration in The Cancer Genome Atlas (TCGA) prostate cancer samples (Figure 3A). We therefore sought to determine the mechanism by which AR signaling suppresses this intrinsic signaling to avoid engaging innate immunity. By analyzing the RNA-Seq data from both C4-2 and Myc-CaP cell lines treated with ENZ for an extensive period (~60 hours), we found that among the top 5 ENZ-regulated genes, *IKBKE* (mouse *Ikbke*) was the only gene whose expression was commonly upregulated by ENZ in both C4-2 and Myc-CaP cells (Figure 3, B and C). Of note, among these ENZ-responsive genes, only the expression of *IKBKE* was uniquely suppressed by acute treatment (24 hours) of dihydrotestosterone (DHT) in both C4-2 and Myc-CaP cell lines (Figure 3, D and E), suggesting that *IKBKE* could be a transcription repression target of the AR. Analysis of AR ChIP-Seq data showed that DHT treatment failed to induce AR binding at the top 5 ENZ-affected gene loci except for that of *IKBKE* (Supplemental Figure 3, A–E). We further confirmed that DHT induced AR binding at the *IKBKE* gene locus and that DHT suppressed, but ENZ increased, *IKBKE* mRNA expression in LNCaP cells (Supplemental Figure 3, F–J). We also examined the correlation between the expression of AR signature genes (AR score) and *IKBKE* expression levels in TCGA prostate cancer patient samples. We noticed a negative correlation between the AR score and *IKBKE* mRNA expression in these patient specimens (Figure 3F). RNA-Seq data and RT-qPCR analysis showed that ENZ treatment also largely increased *IKBKE* expression in C4-2 and Myc-CaP cell lines (Figure 3, G–J). Western blot analysis showed that IKKε protein levels increased with ENZ but were reduced with DHT in both C4-2 and Myc-CaP cells (Figure 3, K and L). To further interrogate AR-mediated IKKε suppression, we knocked down AR expression in LNCaP and C4-2 cells with AR shRNAs. AR depletion largely increased IKKε protein expression in both cell lines (Figure 3M). We also transfected AR^–^ PC-3 and DU145 cells with the AR and found that ectopic AR expression substantially suppressed IKKε protein expression (Figure 3N). We further queried TCGA database for *IKBKE* expression in malignant and normal tissues. We calculated the ratio of *IKBKE* expression in each tumor compared with adjacent normal tissue and plotted the data by cancer type. *IKBKE* expression was elevated in the malignant cells versus normal control tissue among the 17 analyzed cancer types except prostate cancer. Specifically, *IKBKE* was significantly downregulated in prostate adenocarcinoma (PRAD) samples relative to normal prostate tissues (Figure 3, O and P). These data suggest that expression of IKKε, a key positive regulator of innate immunity, was suppressed by AR signaling in the human and murine prostate cancer cell lines and patient samples examined.

### AR suppresses IKBKE transcription through cooperative action of an eRNA and HDAC2.

**Figure 4:**
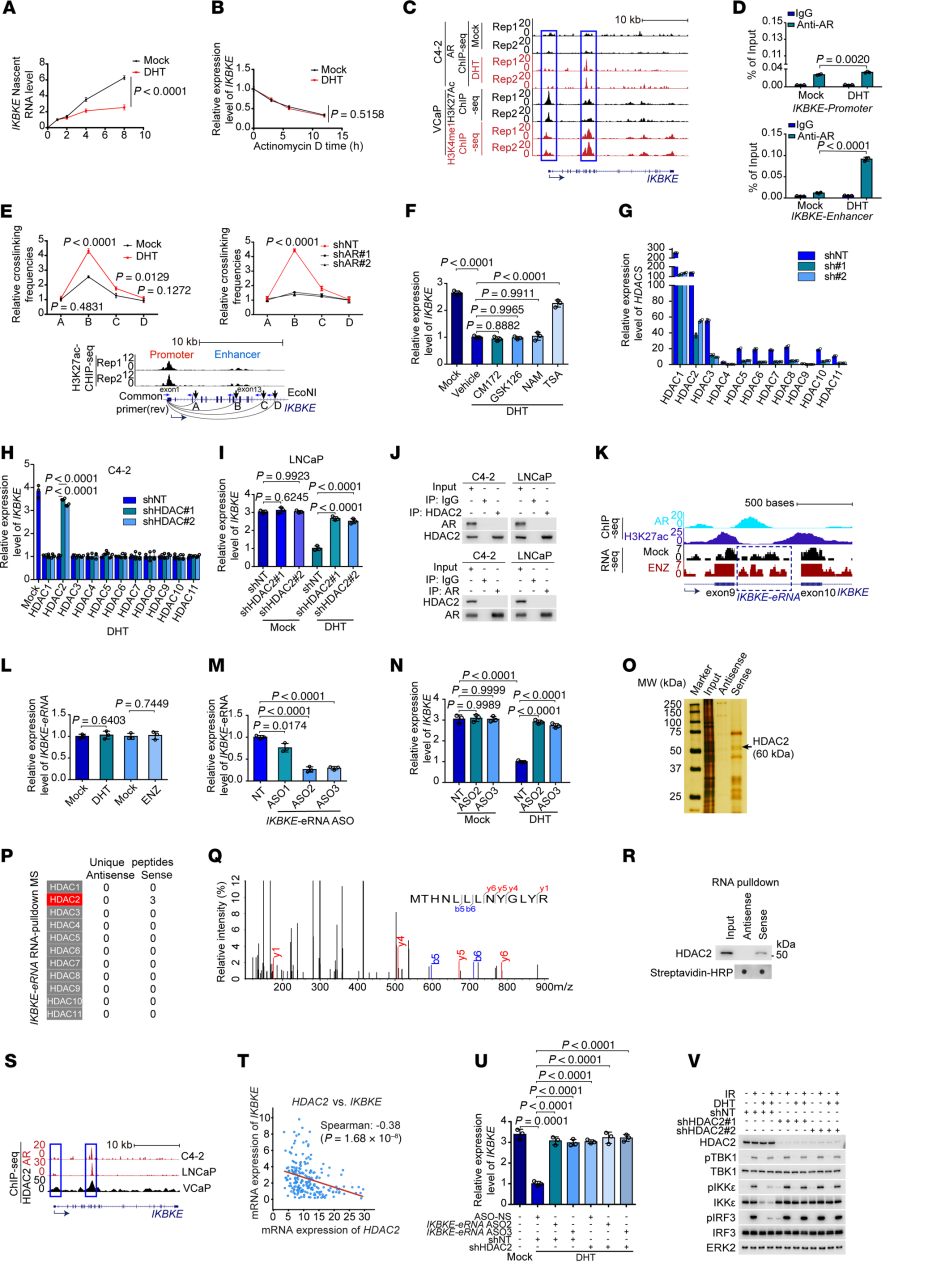
The AR cooperates with HDAC2 to suppress *IKBKE* transcription. (**A**) Nascent RNA synthesis of *IKBKE* mRNA from C4-2 cells. (**B**) Half-life analysis of *IKBKE* mRNA from C4-2 cells. (**C**) UCSC Genome Browser screen shot of *IKBKE* gene locus using AR, H3K27ac, and H3K4me1 ChIP-Seq data. (**D**) ChIP-qPCR analysis of AR occupancy at the *IKBKE* gene promoter and enhancer in C4-2 cells. (**E**) 3C-qPCR analysis of chromatin looping at the *IKBKE* gene locus. (**F**) RT-qPCR analysis of *IKBKE* mRNA in C4-2 cells treated with indicated drugs. (**G**) RT-qPCR analysis of *HDAC* mRNA in C4-2 cells. (**H**) RT-qPCR analysis of *IKBKE* mRNAs in C4-2 cells transfected with HDAC-specific shRNAs. (**I**) RT-qPCR analysis of *IKBKE* mRNA in LNCaP cells. (**J**) Co-IP analysis of the interaction between endogenous AR and HDAC2. (**K**) UCSC genome browser screenshot showing the expression of *IKBKE-e* in C4-2 cells. (**L**) RT-qPCR analysis of *IKBKE-e* expression in C4-2 cells. (**M**) RT-qPCR analysis of *IKBKE-e* expression in C4-2 cells treated with *IKBKE-e*–targeting ASOs. (**N**) RT-qPCR analysis of *IKBKE* mRNA expression in C4-2 cells treated with *IKBKE-e*–targeting ASOs in the presence or absence of DHT treatment. (**O** and **P**) Silver staining of proteins in C4-2 cell lysates pulled down by biotin-labeled *IKBKE-e* (**O**) and MS detection of HDAC family proteins pulled down by biotin-labeled *IKBKE-e* (**P**). (**Q**) Spectrum of a HDAC2 peptide identified by MS. (**R**) Western blot analysis of HDAC2 protein pulled down by a biotin-labeled sense or antisense sequence of *IKBKE-e*. (**S**) UCSC screenshot of AR and HDAC2 ChIP-Seq data in the *IKBKE* gene locus. (**T**) Analysis of the correlation between *IKBKE* and *HDAC2* mRNA expression in TCGA. (**U**) RT-qPCR analysis of *IKBKE* mRNA expression in C4-2 cells treated with *IKBKE-e*–targeting ASOs or HDAC2 shRNA. (**V**) Western blot analysis of the indicated proteins in C4-2 cells infected with lentivirus expressing HDAC2-specific shRNA treated with or without DHT and/or IR (6 Gy). Data are displayed as the mean ± SD of triplicate experiments (**A**, **B**, **D**–**I**, **L**–**N**, and **U**). Significance was determined by 2-tailed *t* test (**A**, **B**, **E**, **G**, **H**, **L**, and **M**), 2-way ANOVA with Tukey’s correction for multiple comparisons (**D**, **N**, and **U**), and 1-way ANOVA with Dunnett’s correction for multiple comparisons (**F** and **I**).

We next sought to determine whether the AR suppresses *IKBKE* mRNA transcription or affects its stability in prostate cancer cells. We treated cells with the transcription inhibitor actinomycin D and measured *IKBKE* mRNA expression in the presence or absence of DHT. *IKBKE* transcription was reduced by DHT only in cells without actinomycin D treatment (Figure 4, A and B), indicating that the AR inhibits *IKBKE* mRNA expression at the transcriptional level. Analysis of AR, H3K4me1, and H3K27Ac (2 enhancer histone markers) ChIP-Seq data showed that DHT induced AR binding within a region of the *IKBKE* gene locus where extensive H3K4me1 and H3K27Ac enrichment was detected (Figure 4C), implying a putative enhancer region. A ChIP-qPCR assay confirmed that the AR bound at both the promoter and putative enhancer regions at the basal level, but DHT largely enhanced AR binding at the putative enhancer region (Figure 4D). We further performed a chromatin conformation capture (3C) assay, which demonstrated that DHT enhanced the approximation of the promoter and the putative enhancer at the *IKBKE* gene locus, but this effect was abolished by AR depletion (Figure 4E).

To determine how the AR, generally acting as a transcription activator upon activation by androgens, can transcriptionally repress *IKBKE* gene expression, we treated cells with inhibitors of commonly recognized transcription repressors including G9a (CM172), EZH2 (GSK126), sirtuin (nicotinamide [NAM]), and HDAC (TSA). We found that the HDAC inhibitor TSA, but not the other inhibitors examined, reversed DHT-induced repression of *IKBKE* expression (Figure 4F), suggesting the involvement of HDAC proteins in the repression. We subsequently knocked down all 11 members of the HDAC family individually by shRNAs and found that only HDAC2 knockdown abolished androgenic inhibition of *IKBKE* expression in C4-2 cells (Figure 4, G and H). HDAC2 knockdown also abolished DHT repression of *IKBKE* in LNCaP cells, a different AR prostate cancer cell line (Figure 4I). Next, we sought to determine whether the AR interacts with HDAC2 by performing a reciprocal co-IP assay. We pretreated cell lysates with ethidium bromide prior to the co-IP assay to minimize DNA-mediated indirect interaction of chromatin-associated proteins. Co-IP assays showed that there was no interaction between endogenous AR and HDAC2 in C4-2 or LNCaP cell lines (Figure 4J). This result is not entirely surprising, considering that the AR generally associates with transcription coactivators and acts as a transcription activator.

To further explore the underlying mechanism of *IKBKE* repression, considering that AR-mediated transcription repression often occurs in a gene-specific manner, we postulated that elements in the *IKBKE* gene locus might be crucial. To this end, we analyzed RNA-Seq data in C4-2 cells and noticed that an approximately 450 bp RNA was expressed in the putative AR-bound *IKBKE* gene enhancer, which we termed *IKBKE*-eRNA (or *IKBKE-e*) (Figure 4, C and K). Intriguingly, *IKBKE-e* expression remained unaltered upon treatment with either DHT or antiandrogen ENZ (Figure 4, K and L). To investigate the functional role of *IKBKE-e*, we identified 2 potent *IKBKE-e*–targeting antisense oligonucleotides (ASOs) (Figure 4M). We demonstrated that *IKBKE-e* ASO treatment largely abolished DHT-induced repression of *IKBKE* mRNA (Figure 4N). These findings suggest that *IKBKE-e* may play an imperative role in mediating DHT inhibition of *IKBKE* transcription.

To further explore the mechanism, we performed an RNA-pulldown assay using biotin-labeled *IKBKE-e* followed by mass spectrometry (MS). We demonstrated that among the proteins detected by MS, HDAC2 was the only member of the HDAC family that was pulled down by *IKBKE-e* (Figure 4, O–R). Meta-analysis of ChIP-Seq data revealed that HDAC2 occupation was also detected in the AR-bound *IKBKE* gene enhancer (Figure 4, C and S), although it appeared that HDAC2 was less likely to be involved in the androgen-induced chromatin looping in this locus (Supplemental Figure 3, K and L). ChIP-qPCR analysis showed that HDAC2 binding at the *IKBKE* locus was largely diminished by depletion of *IKBKE-e* eRNA (Supplemental Figure 3M). Meta-analysis of prostate cancer TCGA database revealed a negative correlation between *HDAC2* and *IKBKE* mRNA expression (Figure 4T). *IKBKE-e* depletion by specific ASOs, *HDAC2* shRNAs, or a combination of these abolished DHT repression of *IKBKE* expression (Figure 4U), implying that *IKBKE* repression caused by *IKBKE-e*– or HDAC2 could be mediated through the same pathway. Importantly, depletion of HDAC2 also abolished AR-mediated inhibition of IKBKE expression and phosphorylation of IRF3, a downstream phosphorylation target of IKKε in the presence of radiation (Figure 4V). These data indicate that expression of both *IKBKE-e* and HDAC2 are important for AR-mediated repression of *IKBKE* mRNA in prostate cancer cells.

### AR-targeted therapy restores IKKε expression and enables IFN signaling in prostate cancer cells.

**Figure 5:**
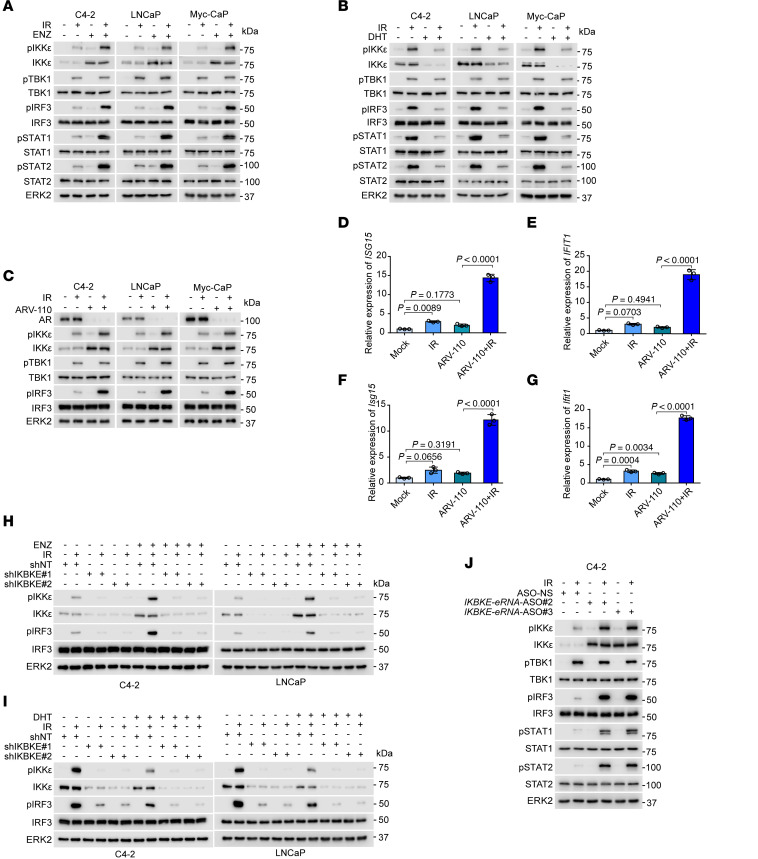
Restored IKKε expression by AR-targeted therapy enables IFN signaling in IR cotreated prostate cancer cells. (**A**) Western blot analysis of the indicated proteins in C4-2, LNCaP, and Myc-CaP cells treated with vehicle or ENZ (10 μM, 48 hours) and then treated with or without IR (6 Gy). (**B**) Western blot analysis of the indicated proteins in C4-2, LNCaP, and Myc-CaP cells treated with CSS medium for 3 days and supplemented with vehicle or DHT (10 nM, 24 hours), and then treated with or without IR (6 Gy). (**C**) Western blot analysis of the indicated proteins in C4-2, LNCaP, and Myc-CaP cells treated with or without ARV110 (5 μM, 24 hours) followed by IR (6 Gy). (**D**–**G**) RT-qPCR analysis of *ISG15* and *IFIT1* mRNA expression in C4-2 (**D** and **E**) and Myc-CaP (**F** and **G**) cells treated with or without ARV110 (5 μM) and followed with or without IR (6 Gy). (**H**) Western blot analysis of the indicated proteins in C4-2 and LNCaP cells transfected with shNT or shIKBKE, then treated with or without ENZ (10 μM) and followed with or without IR (6 Gy). (**I**) Western blot analysis of the indicated proteins from C4-2 and LNCaP cells transfected with shNT or shIKBKE cultured in CSS medium supplemented with DHT (10 nM, 24 hours) and/or IR (6 Gy). (**J**) Western blot analysis of the indicated proteins from C4-2 cells transfected with a nonspecific (NS) control ASO or *IKBKE-e*–specific ASOs followed with or without IR (6 Gy). Data are displayed as the mean ± SD of triplicate experiments (**D**–**G**). Significance was determined by 1-way ANOVA, and Tukey’s correction was applied for multiple comparisons (**D**–**G**).

We next examined whether clinically viable antiandrogen treatments may help induce intrinsic IFN signaling in prostate cancer cells. We treated C4-2, LNCaP, and Myc-CaP prostate cancer cell lines with ENZ and/or IR and measured IRF3 phosphorylation. IRF3 phosphorylation was increased minimally by ENZ or IR alone, but was substantially increased by the combination in these cell lines (Figure 5A). To further characterize the feed-forward process of IFN signaling, we measured STAT1 and STAT2 (STAT1/STAT2) phosphorylation by Western blotting. As expected, IR/ENZ markedly increased STAT1/STAT2 phosphorylation, confirming that IFN signaling was significantly increased by combined IR/ENZ (Figure 5A). We also examined the effect of androgen deprivation on IFN signaling in prostate cancer cells. We grew these cell lines with media containing charcoal-stripped bovine serum (CSS) for 3 days followed by DHT treatment and/or radiation (6 Gy). We demonstrated that DHT substantially diminished IR-induced phosphorylation of IRF3, STAT1, and STAT2 proteins (Figure 5B). These findings suggest that androgen signaling is an essential suppressor of intrinsic innate immunity in prostate cancer cells in response to radiation.

AR degraders such as ARV-110 are emerging as an effective ARPI for research use and clinical testing (35, 36). We treated C4-2, LNCaP, and Myc-CaP cell lines with IR and/or ARV-110 and measured the activation of PAMP mediators including IKKε and IRF3. ARV-110 upregulated IKKε expression and combined IR/ARV-110 largely increased IKKε and IRF3 protein phosphorylation (Figure 5C). ARV-110 plus IR enhanced mRNA expression of IFN signaling genes such as *ISG15* and *IFIT1* in both C4-2 and Myc-CaP cell lines (Figure 5, D–G). Furthermore, we showed that knockdown of *IKBKE* by 2 independent shRNAs diminished IKKε and IRF3 phosphorylation induced by IR alone or in combination with ENZ in C4-2 and LNCaP cells cultured in regular (androgen-containing) medium (Figure 5H). In contrast, DHT treatment decreased IKKε and IRF3 protein phosphorylation induced by IR in C4-2 and LNCaP cells cultured in androgen-depleted medium, but such difference was abolished in *IKBKE*-knockdown cells (Figure 5I). We also depleted *IKBKE-e* using specific ASOs and demonstrated that *IKBKE-e* depletion augmented IKKε and IRF3 phosphorylation in a manner similar to AR inhibition by ENZ (Figure 5J). Collectively, these data indicate that restored expression of IKKε is important for ARPI/IR dual-treatment–induced activation of IFN signaling and an innate immune response in prostate cancer cells.

### The importance of the dsRNA recognition proteins RIG-I and MDA5 in ENZ/IR-induced activation of the innate immune response.

**Figure 6:**
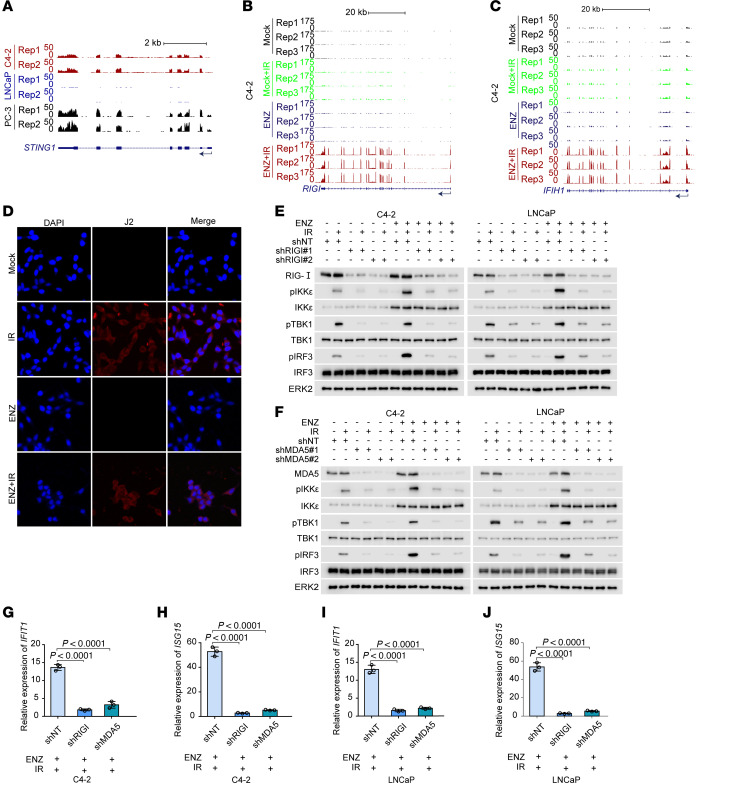
IR and antiandrogen dual treatment activates IFN signaling via RIG-I and MDA5. (**A**) UCSC Genome Browser screenshot of the *STING1* gene using RNA-Seq data from C4-2, LNCaP, and PC-3 cells. (**B** and **C**) UCSC Genome Browser screenshot of *RIGI* (**B**) and *IFIH1* (**C**) using RNA-Seq data from C4-2 cells treated with ENZ (10 μM, 48 hours), IR, or their combination. (**D**) IF analysis of dsRNA production using J2 antibody in C4-2 cells treated with ENZ (10 μM, 48 hours), IR, or their combination. Original magnification, ×40. (**E** and **F**) Western blot analysis of the indicated proteins in C4-2 and LNCaP cells infected with lentivirus for shNT (nontargeting) or shRIGI (**E**) or shMDA5 (**F**) treated with or without IR (6 Gy) and/or ENZ (10 μM) for 48 hours. (**G**–**J**) RT-qPCR analysis of *IFIT1* and *ISG15* mRNA expression in C4-2 and LNCaP cells transfected with the indicated shRNAs and treated with ENZ or IR. Data are displayed as the mean ± SD of triplicate experiments (**G**–**J**). Significance was determined by 1-way ANOVA, and Dunnett’s correction was applied for multiple comparisons (**G**–**J**).

To determine the upstream regulators of the IKKε/IRF3 signaling in androgen-responsive cells, we first examined the expression of *STING1* mRNA, which encodes STING protein, a key mediator of the dsDNA-induced innate immune response. Analysis of RNA-Seq data showed that *STING1* mRNA expression was lower or minimally detectable in AR^+^ C4-2 and LNCaP cell lines compared with expression in the AR^–^ cell line PC-3 (Figure 6A). *STING1* mRNA expression was unchanged in LNCaP cells or even downregulated in C4-2 cells following ENZ treatment (Supplemental Figure 3N). In contrast, mRNA expression of *RIGI* and *IFIH1*, which encode the dsRNA sensor proteins RIG-I and MDA5, respectively, was largely upregulated 6 hours (short-term) after ENZ/IR treatment (Figure 6, B and C), consistent with the activation of the innate immune response under the same treatment conditions (Figure 5A). The findings prompted us to hypothesize that the innate immune activation caused by dual therapy is likely mediated by the dsRNA-sensing pathway. In support of this notion, dsRNAs were detected in C4-2, LNCaP, and Myc-CaP cell lines upon IR treatment, regardless of ENZ exposure (Figure 6D and Supplemental Figure 4, A and B). We also performed similar experiments using poly(I:C) to engage PAMPs. We showed that ENZ enhanced, but DHT diminished, poly(I:C)-induced phosphorylation of IKKε, IRF3, STAT1, and STAT2 in C4-2, LNCaP, and Myc-CaP cell lines (Supplemental Figure 4, C and D). Although no obvious increase in RIG-I and MDA5 expression at the protein level under treatment with IR/ENZ for a short period of time (6 hours) (Figure 6, E and F), which was different from the effect of longer treatment durations (48 hours) on the expression of these mRNAs (Figure 6, B and C), we found that knockdown of either RIG-I or MDA5 abolished IR/ENZ-induced phosphorylation of IKKε and IRF3 proteins and mRNA expression of innate immune response genes (Figure 6, E–J). These findings highlight the importance of the dsRNA sensor proteins RIG-I and MDA5 in ENZ/IR-induced activation of the innate immune response.

### Dual radiation and antiandrogen sensitize prostate cancer cells to ICI treatment in mice.

**Figure 7:**
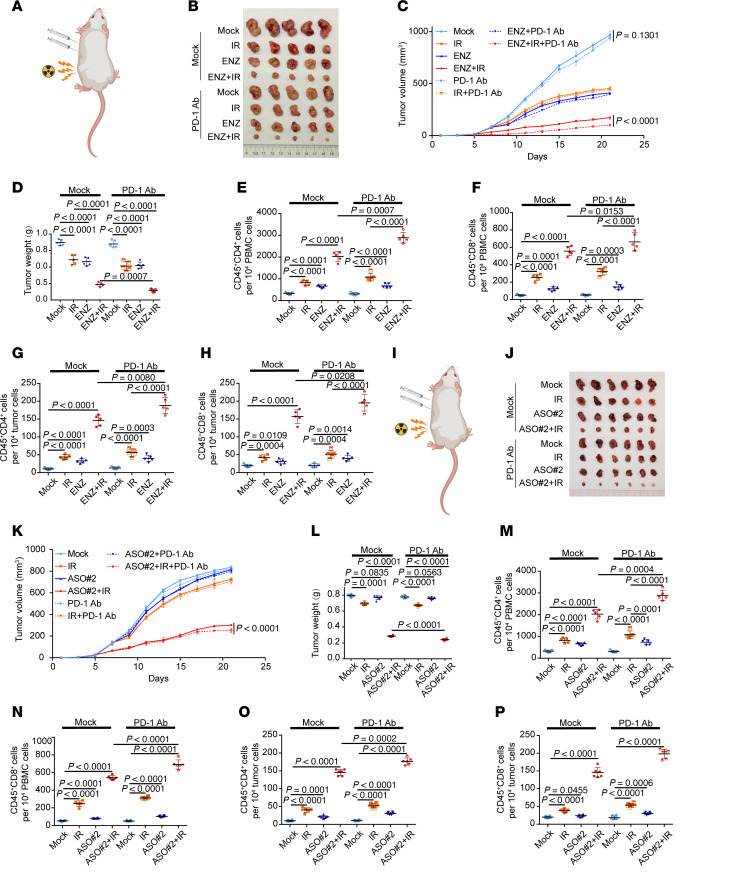
IR and antiandrogen dual treatment sensitizes prostate cancer cells to ICI therapy. (**A**) Illustration of the mouse treatment. Myc-CaP cells were injected s.c. into the right flank of mice. After 7 days, tumors were treated with or without IR (6 Gy), ENZ (10 mg/kg), and/or InVivoMAb anti–mouse PD-1 antibody (10 mg/kg). (**B**–**D**) Tumors from mice in each group at day 21 were harvested and photographed (**B**). Tumor growth was measured every other day for 21 days. Tumor volumes at each time point were measured (**C**), and dot plots show tumor weights for mice in each group at day 21 (**D**). (**E**–**H**) CD4^+^ and CD8^+^ T cells in PBMCs (**E** and **F**) and tumors (**G** and **H**) were analyzed by FACS. (**I**) Illustration of the mouse treatment. C4-2 cells were injected s.c. into the right flank of BRGSF-HIS mice. After 7 days, tumors were treated with or without IR and *IKBKE*-eRNA ASO (10 mg/kg). (**J**–**L**) Tumor growth was measured every other day for 21 days. Tumor growth over the 21-day period (**K**). Tumors in each group at day 21 were harvested and photographed (**J**), and tumor weights were measured (**L**). (**M** and **N**) CD45^+^CD4^+^ (**M**) and CD45^+^CD8^+^ (**N**) lymphocytes in PBMCs of mice treated as indicated were analyzed by FACS. (**O** and **P**) Tumor tissues from mice treated as indicated were digested, and infiltrated CD45^+^CD4^+^ (**O**) and CD45^+^CD8^+^ (**P**) lymphocytes were analyzed by FACS. Data are displayed as the mean ± SD (*n* = 5) (**C**–**H** and **K**–**P**). Significance was determined by 2-way ANOVA (**C**–**H** and **K**–**P**).

We have observed that ARPI and IR combination induced innate immune signaling, increased anticancer T cell infiltration, and conferred long-term survival in metastatic CRPC. This dual treatment appears to operate through increased IKKε availability and phosphorylation in prostate cancer cells. As noted above, ICI treatment alone was performed poorly in CRPC. Next, we sought to determine whether cotreatment of radiation and antiandrogen sensitizes prostate cancer cells to ICI therapy in mice. As expected, ENZ/IR combination resulted in much greater inhibition of Myc-CaP tumor growth compared with ENZ or IR treatment alone and, importantly, cotreatment of anti–PD-1 antibody with ENZ and IR further decreased tumor growth, although anti–PD-1 antibody alone had little or no effect on tumor growth (Figure 7, A–D). Compared with ENZ/IR, trio treatment with IKBKE-ε ASO/IR/anti–PD-1 antibody not only caused further elevation of CD4^+^ and CD8^+^ T cells including memory T cells (CD4^+^CD45RO^+^ and CD8^+^CD45RO^+^) in PBMCs of mice, but also induced much higher-level infiltration of CD4^+^ and CD8^+^ T cells in tumors (Figure 7, E–H, and Supplemental Figure 5, A–F). This result is consistent with the findings from T cell transplant assays and CD4^+^ and CD8^+^ T cell depletion studies (Supplemental Figures 6 and 7). In agreement with the effects seen in C4-2 and LNCaP cells in vitro (Figure 6, E–J), we found that knockdown of RIG-I or MDA5 largely diminished ENZ/IR treatment–induced inhibition of Myc-CaP murine tumors in immunocompetent mice (Supplemental Figure 8). As treatment of *IKBKE-e* ASOs largely enhanced the IR-induced innate immune response in cultured cells (Figure 5J), we next sought to determine whether *IKBKE-e* ASO in combination with IR induces an innate immunity response in mice that could be harnessed to overcome ICI resistance in murine prostate tumors. Once the Myc-CaP tumors were established in recipient mice, we treated the mice with IR, *IKBKE-e* ASO, and/or PD-1–inhibitory antibody. As expected, treatment with anti–PD-1 antibody, IR, or *IKBKE-e* ASO alone did not result in a substantial reduction in tumor growth, recapitulating the failure of immunotherapy, IR, or ARPI alone in CRPC observed in multiple clinical trials. However, trio treatment with *IKBKE-e* ASO/IR/PD-1 antibody led to a much greater reduction in tumor size (Figure 7, I–L). We also measured CD4^+^ and CD8^+^ lymphocytes in PBMCs and tumors in this model. *IKBKE-e* ASO/IR/PD-1 antibody treatment significantly increased the immune response in PBMCs and the tumor microenvironment (Figure 7, M–P). Together, these data suggest that restoring *IKBKE* expression by ARPI or *IKBKE-e* ASO treatment in combination with IR could unlock a population of T cells that can be mobilized to enhance immunotherapy.

## Discussion

Despite the fact that immune monotherapy has been an effective treatment in many cancer types, prostate cancer remains as an immunologically “cold” cancer (37). Cancer cell–targeting plus immune therapies are emerging as a viable therapeutic strategy for various cancers, but ARPI in combination with ICI is also ineffective in prostate cancer, at least in castration-resistant settings (12, 38). Our findings demonstrate that the combination of IR and ARPI resulted in enhanced immune activation and increased tumor-reactive T cell responses in prostate cancer cells in patients and mice. We also show that ARPI/IR combination further enhanced anticancer efficacy of ICI in a mouse model. Our study provides molecular insights into the interplay between radiation, AR signaling, and immune responses, highlighting the potential for combination therapies to elicit robust antitumor immune responses in metastatic CRPC.

It has been shown previously that primary prostate cancers that are not treated with hormones have limited numbers of infiltrated immune cells (39, 40). However, the propensity of immune tolerance in prostate cancer cannot be simply explained by the previous findings that the AR influences innate and adaptive immune systems by systematically suppressing neutrophil function and T and B cell development, as demonstrated in mice, and that AR activity in T cells limits ICI efficacy in patients with metastatic CRPC (9, 11). In contrast to these reports, we identify a previously uncharacterized activity of the AR that suppressed anticancer immunity by limiting the innate immune response in prostate cancer cells. Our analysis of scRNA-Seq data in PBMCs of patients with tumors treated with radiation revealed that good responders had a greater number of cytotoxic CD8^+^ T cells. These data imply that radiation of tumors could trigger activation of anticancer immunity in patients. This could be due to the death of tumor cells or the release of certain factors such as cytokines from irradiated tumor cells, thereby resulting in the systematic activation of the immune system in blood. Similar mechanisms could also occur in mice, providing a plausible explanation for the T cell infiltration in the contralateral tumors without IR treatment.

Mechanistically, our study revealed that the key innate immune-regulatory gene *IKBKE* was uniquely downregulated in cancer cells compared with normal cells in primary prostate cancers of patients in TCGA, but not in the 16 other cancer types examined, implying that the IKKε-mediated innate immune response, including IFN signaling and expression of antigen presentation machinery genes, was compromised in hormone-naive prostate cancer. Importantly, we show that androgen-mediated repression of *IKBKE* was reversible and that *IKBKE* expression could be derepressed following ARPI treatment, including ENZ or AR degraders. Thus, restored expression of IKKε in hormone-responsive prostate cancer cells provides a plausible explanation, at least in part, for the previous observations that androgen deprivation therapy induces immunogenic changes in the tumor microenvironment of hormone-sensitive prostate cancers, including decreased tolerance and clonal expansion of effector T cells and stimulation of the antigen-specific adaptive immune response among others (41–44). On the basis of our findings, we envision that the strategy of radiation plus AR signaling inhibition (leading to IKKE upregulation) could be effective in treatment-naive metastatic prostate cancer and that radiation in combination with an AR degrader could be one of the viable options. Similarly, IR could also be sufficient to induce a robust immune response via IRF3 phosphorylation downstream of dsRNA recognized by RIG-I/MDA5 in AR^–^ cancers in which IKKε is readily expressed.

**Figure 8:**
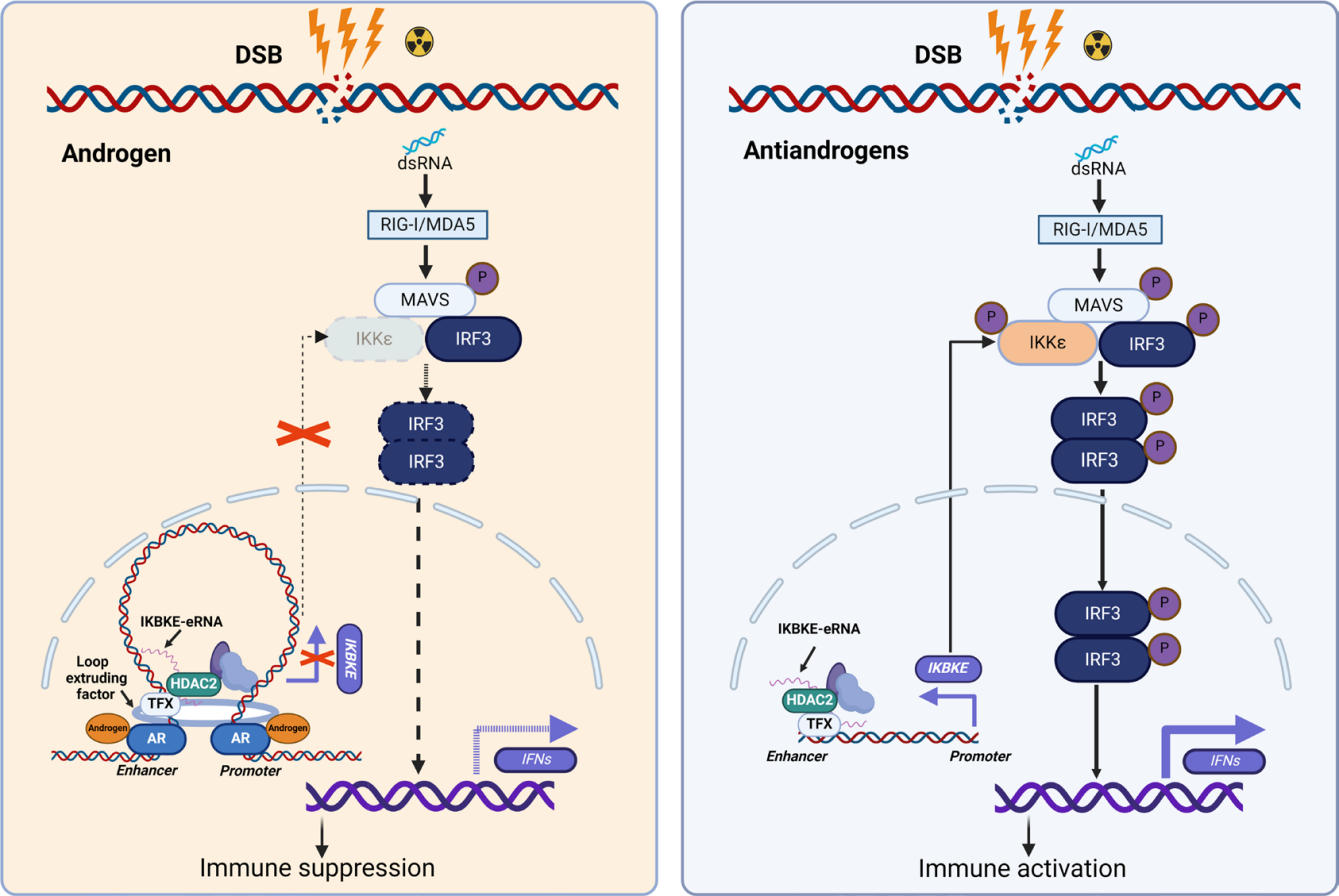
A hypothetical model. Left: Radiation treatment of prostate cancer cells induces the production of dsRNAs. In androgen-stimulated prostate cancer cells (low or no *STING1* expression), the androgen-activated AR promotes the repression of *IKBKE* mRNA by inducing chromatin looping between a putative enhancer bound by *IKBKE*-eRNA–tethered HDAC2 and the promoter of the *IKBKE* gene, resulting in the transcriptional repression of *IKBKE* mRNA and downregulation of IKKε protein, a key effector protein mediating dsRNA-induced IRF3 phosphorylation and the innate immune response. Right: Upon treatment of antiandrogens such as ENZ, AR-mediated transcriptional repression of *IKBKE* is reversed by the interruption of AR-mediated chromatin looping between the *IKBKE* gene promoter and enhancer, permitting radiation-generated dsRNA to activate intrinsic innate immune signaling in prostate cancer cells. DSB, double-stranded DNA break; MAVS, mitochondrial antiviral-signaling protein.

It is well documented that the androgen-activated AR causes both up- and downregulation of target genes (8, 45–47). AR-induced gene expression can be explained by its interaction with transcription coactivators such as CBP and p300 and its role in facilitating the chromatin looping between the gene promoter and enhancer(s) through its binding at both regions (48, 49). However, the molecular mechanism underlying AR-mediated gene repression remains far from elucidated, given that the selective downregulation of a subset of genes in the genome caused by the AR cannot be simply explained by its binding of transcriptional corepressors with a general role in gene repression. In support of this notion, we provide evidence that the AR has no interaction with the transcription corepressor protein HDAC2. In contrast, we show that upon activation by androgens, AR promoted the chromatin looping between the HDAC2-bound enhancer and the promoter of the *IKBKE* gene. Given these observations, we envisage a hypothetical model of AR-mediated repression of *IKBKE* transcription (Figure 8) in which androgen stimulation activates the AR, which in turn results in chromatin looping between the promoter and the HDAC2-bound enhancer at loci of target genes such as *IKBKE*, thereby causing the transcriptional repression of *IKBKE* and suppression of innate immunity (Figure 8). Upon antiandrogen treatment, chromatin looping–mediated gene repression is abolished, leading to *IKBKE* derepression and innate immune activation (Figure 8). Our findings not only provide a plausible explanation for how the transcriptionally active AR mediates repression of its target genes such as *IKBKE*, but also reveal a potential general mechanism that could be applicable to the gene repression mediated by other transcription activators (Figure 8).

Collectively, the findings from this study shed light on the potential of the combination of ARPI and radiation therapy to improve the anticancer efficacy of immunotherapy in prostate cancer. We not only reveal an intrinsic role of the, AR in suppressing innate immunity in malignant epithelial cells of the prostate, but mechanistically, we also identify *IKBKE* eRNA and HDAC2 as 2 key effectors in mediating the immunosuppressive activity of the AR in prostate cancer. Our observations suggest that rejuvenating IKKε signaling via targeting of *IKBKE* eRNA in combination with radiation could be an effective means to trigger anticancer immunity in immunologically “cold” cancers such as prostate cancer. Further studies are needed to validate these findings and explore their translation to clinical practice.

## Methods

### Sex as a biological variable

Our study exclusively examined male mice because prostate cancer is only relevant in male individuals.

Mice were housed in 22°C at 55% humidity on average, with a 12-hour light/12-hour dark cycle and access to food and water. The maximum volume of the grafts was limited within 1 cm^3^ according to the ethics requirements.

### Cell culture and transfection

LNCaP, C4-2, Myc-CaP, PC-3, DU145, and 293T cell lines were purchased from the American Type Culture Collection (ATCC). LNCaP, C4-2, PC-3, and DU145 cell lines were cultured in RPMI 1640 cell culture medium (Corning) with 10% FBS, together with 100 μg/mL streptomycin and 100 U/mL penicillin. Myc-CaP and 293T cells were cultured in DMEM cell culture medium (Corning) with 10% FBS. Mycoplasma contamination was regularly examined using the Lookout Mycoplasma PCR Detection Kit (MilliporeSigma). Transfections were performed using Lipofectamine 2000 (Thermo Fisher Scientific). Control shRNAs and gene-specific shRNAs for *AR*, *IKBKE*, *RIGI*, *IFIH1*, and *HDAC* genes were purchased from MilliporeSigma, and sequence information for each shRNA is provided in Supplemental Table 1.

### Design and screening of ASOs

The ASOs used in this study are single-strain DNA and were designed and synthesized by Integrated DNA Technologies Inc. (IDT) based on the complementary sequence of *IKBKE-e* with a phosphorothioate backbone and 2′-O-methoxyethylribose (MOE) modification on the flanking 6 nucleotides. ASOs were transfected into C4-2 cells using polyethylenimine (PEI), similarly to the in vivo experiments. The ASO sequences are listed in Supplemental Table 1.

### Patient information

We previously conducted a longitudinal study at the Mayo Clinic involving 84 patients with oligometastatic CRPC from August 2016 to December 2019 (NCT02816983) (23). In brief, patients with histologically confirmed prostate cancer with 3 or fewer lesions identified on choline PET/CT with castrate levels of testosterone (<50 mg/dL) underwent continued ADT and stereotactic body radiotherapy on all visible sites of metastasis. Patients were followed for OS and for local, distant, and biochemical PFS. Patients underwent blood draws to collect PBMCs before and after treatment. Flow cytometry was performed to identify T cell subpopulations with CCR7-BV650, CD11a-APC, CD45RA, CD8-PE-Cy7, Ki-67-BV421, and PD-1 FITC. Statistical comparisons were performed by Cox proportional hazards modeling. All patients from Changhai Hospital in Shanghai were diagnosed with locally advanced prostate cancer with a Gleason score of 8 or higher. These patients underwent 3-month ADT plus approximately 4–8 weeks of radiation treatment (irradiated 5 times with each fraction ranging from 1.8–2 Gy) with 72–80 Gy in total.

### scRNA-Seq, bulk RNA-Seq, and data analysis

Live cells were washed twice in 1× PBS plus 0.04% BSA and immediately submitted to the Genome Analysis Core (GAC) at the Mayo Clinic for single-cell partitioning. The cells were first counted and measured for viability using the Vi-Cell XR Cell Viability Analyzer (Beckman-Coulter). The barcoded Gel Beads were thawed from –80°C, and the cDNA master mix was prepared according to the manufacture’s instruction for the Chromium Next GEM Single Cell 5′ Library and Gel Bead Kit (10x Genomics). A volume of live cells was mixed with the cDNA master mix according to the desired number of cells to be captured for each sample. A concentration of 500,000 cells/mL or better for each sample was required for the standard targeted cell recovery of 5,000 cells. The cell suspension and master mix, thawed Gel Beads, and partitioning oil were added to a Chromium Single Cell G chip. The filled chip was loaded into the Chromium Controller, where each sample was processed, and the individual cells within the sample were partitioned into uniquely labeled gel beads in emulsion (GEMs). The GEMs were collected from the chip and taken to the bench for reverse transcription, GEM dissolution, and cDNA clean-up. The full-length cDNA was amplified and separated by size selection. The resulting cDNA was a pool of uniquely barcoded molecules used to generate 5′ gene expression libraries and enriched T cell receptor (TCR) libraries. During library construction, standard Illumina sequencing primers and a unique i7 Sample index (Single Index Kit T Set A, 10x Genomics) were added to each cDNA pool (gene expression and TCR).

scRNA data were processed using the CellRanger (version 7.0.1) “count” command and the GRCh38 human genome reference. The CellRanger “multi” pipeline was used to aggregate and normalize different samples. Downstream analyses were conducted using Seurat (version 4.3.2) following standard workflows. Cells with fewer than 200 detected genes, more than 5,000 detected genes, or mitochondrial gene content exceeding 15% were excluded. Cell-type annotation was performed using scMayoMap (50), based on the curated cell type marker database. The annotation was further verified with canonical marker genes as previously reported (51, 52). The list of marker genes used for cell-type annotation is provided in Supplemental Table 2. Contaminated erythrocytes were identified and removed prior to further analysis.

Bulk RNA-Seq libraries from total RNA were generated using the Illumina TruSeq RNA preparation kit following the standard protocol. The libraries were sequenced as 51 nt paired-end reads at 1 sample per lane of an Illumina HiSeq 2500, generating an average of 265 million reads per sample. All reads generated from C4-2 were aligned to the human reference genome (GRCh38/hg38) using STAR (2.5.2b). Similarly, mouse RNA-Seq reads were aligned to the mouse reference genome (GRCm38/mm10). Gene expression counts were generated using the RSeQC’s “FPKM_count.py” command. Differentially expressed genes were analyzed using DESeq2, with a cutoff of log_2_ (fold change) = 1 and a *P* value of less than 0.001. GO analysis was performed using the Molecular Signatures Database (MSigDB).

### RT-qPCR

Total RNA was isolated using TRIzol reagent (Ambion, Thermo Fisher Scientific) and reverse transcribed into complementary DNA by utilizing the GoScript Kit (Promega). SYBR Green Master Mix (Bio-Rad) and the CFX96 Real-Time System (Bio-Rad) were used to conduct RT-qPCR according to the manufacturer’s instructions. The 2 ΔCt method was used to quantitate fold changes by normalizing to the internal control GAPDH, and data are presented as the mean ± SD. Primer sequence information for qPCR is provided in Supplemental Table 3.

### Multiplexing IHC

For each patient, a representative formalin-fixed, paraffin-embedded (FFPE) slice was cut and selected for multiplexing IHC (mIHC). Staining was performed using the following antibodies, including CD8+ T cell marker (CD8α [D4W2Z] XP Rabbit mAb, 98941, Cell Signaling Technology), CD4+ T cell marker (Anti-CD4 antibody EPR6855, ab133616, Abcam), DC cell marker (CD11c [D1V9Y] Rabbit mAb, 97585, Cell Signaling Technology), and NK cell marker (NCAM1 (CD56 [E7X9M] XP Rabbit mAb, 99746, Cell Signaling Technology). mIHC staining was performed using the Opal Polaris 7-color IHC kit (NEL861001KT, Akoya Biosciences). All procedures were performed under the guidance of the manufacturer. All sections were baked, deparaffinized, rehydrated, and then subjected to antigen retrieval. The tissue was incubated with a primary antibody, followed by Opal polymer HRP incubation and Opal signal generation. Subsequently, the antibody was stripped again by microwave treatment, and a new round of staining was initiated until all 4 markers were labeled. After staining, the nuclei were stained with DAPI, and the slides were sealed with mounting medium to prevent fluorescence quenching. The stained slides were scanned and visualized on a Vectra Polaris multispectral microscope system (Akoya Biosciences). All steps were batched.

### Generation and treatment of prostate cancer graft tumors in mice

#### Animal study I.

Six-week-old FVB male mice (The Jackson Laboratory) were used in the study. Mice were s.c. injected with Myc-CaP cells (2 × 10^6^) mixed with Matrigel mixture (1× PBS: Matrigel (BD Biosciences) = 1:1). Fifteen days after cell injection, the mice received radiation treatment with or without ENZ (10 mg/kg). Mice were euthanized and tumor grafts were excised at the end of treatment.

#### Animal study II.

Six-week-old FVB male mice (The Jackson Laboratory) were used in the study. Mice were s.c. injected with Myc-CaP cells (2 × 10^6^) mixed with Matrigel mixture (1× PBS: Matrigel [BD Biosciences] = 1:1). Fifteen days after cell injection, the mice received radiation treatment with or without InVivoMAb anti–mouse PD-1 (BioXCell, BE0273) (10 mg/kg) or ENZ (10 mg/kg) treatment. Mice were euthanized and tumor grafts were excised at the end of the treatment.

#### Animal study III.

For *IKBKE-e* ASO treatment of human prostate cancer cells in mice, 13-week-old BRGSF-HIS male mice (genOway) were used. Mice were s.c. injected with C4-2 cells (2 × 10^6^) mixed with Matrigel mixture (1× PBS; Matrigel = 1:1). At 15 days after cell injection, the mice received radiation treatment with or without ASO no. 2 (10 mg/kg) or were given pembrolizumab (10 mg/kg). Mice were euthanized and tumor grafts were excised at the end of treatment.

#### Animal study IV.

For T cell transplantation studies, PBMCs were collected using Ficoll (MilliporeSigma, F4375) from different groups of Myc-CaP tumor–bearing FVB mice (GemPharmatech) that had been mock treated or treated with ENZ, IR, or ENZ/IR in combination with anti–PD-1 antibody for 21 days. PBMCs (5 × 10^6^ cells/mouse) isolated from the treated mice were transferred into SCID mice by tail vein injection. After 7 days, mice were s.c. injected with Myc-CaP cells (2 × 10^6^) mixed with Matrigel mixture (1 × PBS: Matrigel = 1:1). After 15 days of culturing, with or without IR, ENZ (10 mg/kg) and InVivoMAb anti–mouse PD-1 (10 mg/kg), mice were euthanized and tumor grafts were excised at the end of treatment.

#### Animal study V.

Following the protocols reported previously (53–55), we depleted CD4^+^ and CD8^+^ T cells using anti-CD4 or anti-CD8 antibodies in PBMCs from Myc-CaP tumor–bearing FVB mice (GemPharmatech) treated with ENZ, IR, and anti–PD-1 antibody by i.p. injection of 400 μg anti-CD4 antibody (clone GK1.5, BioXCell) and 400 μg anti-CD8 antibody (clone 2.43; BioXCell) twice weekly.

### Radiation treatment of cell lines

Fresh cell culture medium was replaced to ensure good cell culture conditions. IR apparatus was preheated, and a suitable position was selected to adjust the radiation dose to 6 Gy. Cells were taken from the incubator,half of the culture medium was discarded, and the cell culture dish was placed on the platform of the IR apparatus. After taking self-protection measures and confirming that the machine door was closed, IR was performed for radiation therapy. Following radiotherapy, cells were continuously cultured in a 37°C incubator prior to harvesting.

### Western blotting and antibodies

Cells were harvested and lysed by modified RIPA buffer with 1% protease inhibitor mixture. Protein concentration was determined using DC protein assay reagent (Bio-Rad). Samples were mixed with dithiothreitol and loading buffer and boiled at 100°C for 5 minutes. Samples were subjected to SDS-PAGE (Bio-Rad) separation, and the gels were further transferred onto nitrocellulose (NC) membranes (Thermo Fisher Scientific). The NC membrane was blocked with 5% milk (Bio-Rad) for 1 hour at room temperature and incubated with the indicated primary antibodies at 4°C overnight. The next day, the NC membranes were washed 3 times with 1× TBST for 5 minutes each time and incubated with a matched secondary antibody for 1 hour at room temperature. Then, the membranes were washed 3 times with 1× TBST for 5 minutes each time. The protein bands were developed with SuperSignal West Pico Luminal Enhancer Solution (Thermo Fisher Scientific) on autoradiography films (HyBlot). Information on the antibodies used for Western blotting, co-IP, and ChIP is provided in Supplemental Table 4.

### Co-IP assay

Cells were collected and washed with 1× PBS twice, followed by cell lysis in IP buffer (0.5% NP-40, 20 mM Tris-HCl, pH 8.0, 10 mM NaCl, 1 mM EDTA) supplemented with protease inhibitors (MilliporeSigma). For the ethidium bromide (EtBr) treatment, cell lysate was incubated with 50 μg/mL EtBr for 30 minutes at 4°C prior to IP. Anti-AR or anti-HDAC2 antibodies (2 μg) were added to the cell lysate and incubated with Protein A/G Beads (MilliporeSigma) overnight. The beads were washed and boiled with protein-loading dye (Bio-Rad) for further analysis by Western blotting.

### ChIP-coupled qPCR

ChIP was performed as described previously (56). In brief, chromatin was crosslinked for 10 minutes at room temperature with 10% formaldehyde/PBS solution added to the cell culture medium. Crosslinked chromatin was then sonicated, diluted, and immunoprecipitated with Protein G Plus Agarose Beads (Bio-Rad) prebound with antibody at 4°C overnight. Precipitated protein-DNA complexes were eluted, and crosslinking was reversed at 65°C for 18 hours. DNA fragments were purified and analyzed by RT-qPCR. ChIP-qPCR data were analyzed as the percentage of input after normalizing each ChIP DNA fraction’s Ct value to the input DNA fraction’s Ct value.

### Nascent RNA assay

A nascent RNA assay was performed as described previously (57). EU labeling experiments were performed according to the protocol for the Click-iT Nascent RNA Capture kit (Invitrogen, Thermo Fisher Scientific). Briefly, cells were pulsed with 0.5 mM EU, and total RNA was isolated and used in a copper catalyzed click reaction with azide-modified biotin. After this, the nascent transcripts were captured on streptavidin magnetic beads and subjected to cDNA synthesis using the SuperScript VILO cDNA synthesis kit directly on the beads followed by qRT-PCR analysis.

### AR activity score

The AR activity score was calculated on the basis of the 20 AR target genes as described previously (58), including *ABCC4*, *ACSL3*, *ADAM7*, *C1ORF116*, *CENPN*, *EAF2*, *ELL2*, *FKBP5*, *GNMT*, *HERC3*, *KLK2*, *KLK3*, *MAF*, *MED28*, *MPHOSPH9*, *NKX3.1*, *NNMT*, *PMEPA1*, *PTGER4*, and *ZBTB10*. In brief, gene expression values of log_2_ (fragments per kilo base per million mapped reads [FPKM]) of each sample were converted to a *z* score by *z* = (*x* − μ)/σ, where μ is the average log_2_ (FPKM) across all samples of a gene and σ is the standard deviation (SD) of the log_2_ (FPKM). The *z* scores were then summed across all genes for each sample.

### Flow cytometric analysis

Flow cytometric analysis of PBMCs were recovered by centrifugation over Ficoll Paque Plus (Amersham Biosciences). For mouse tissue samples, tumors were cut into small pieces and digested with 1.5 mg/mL collagenase (MilliporeSigma) in DMEM for 1 hour at 37°C. Cells were filtered through a 70 μm nylon strainer and resuspended in RBC lysis buffer (BioLegend) for 5 minutes at room temperature 3 times. Cells were then suspended in PBS with 2% BSA and costained with the following antibodies: CD45, CD4, and CD8 (BioLegend). After incubation with an antibody for 30 minutes, the cells were washed with cold PBS and analyzed on a flow cytometer.

### Statistics

Statistical analyses were performed with a 1-or 2-sided, paired Student *t* test for single comparisons and by 1-way ANOVA with a post hoc test for multiple comparisons unless otherwise specified. *P* values of less than 0.05 were considered statistically significant. All values are expressed as the mean ± SD.

### Study approval

Animal Study I and Study II were approved by the IACUC of the Mayo Clinic. Animal Study III was approved by the Animal Ethics Committee of the First Affiliated Hospital of Zhengzhou University. Animal Study IV and Study V were approved by the Animal Ethics Committee at the First Affiliated Hospital of Zhejiang University School of Medicine. Prostate cancer specimens were obtained from the Mayo Clinic tissue registry. The studies were approved by the IRB of the Mayo Clinic. All specimens were deidentified from patient information. All participants provided written informed consent. This study was conducted in accordance with the guidelines and tenets of the Declaration of Helsinki.

### Data availability

The raw and processed data have been deposited in the NCBI Gene Expression Omnibus (GEO) database (GEO GSE277595), comprising 3 subseries datasets: GSE277590 (scRNA-Seq for patient samples), GSE277591 (C4-2 cell bulk RNA-Seq), and GSE277593 (Myc-CaP cell bulk RNA-Seq). Values for all data points in graphs are reported in the Supporting Data Values file. All other data are available in the main manuscript or the supplemental materials.

## Author contributions

HH, RS, and XL conceived and designed the study. XL, RS, HL, JJO, Y Hou, SSP, YZ, Y He, VRB, JDD, and GF performed experiments and data analysis. XZ and LW analyzed the high-throughput sequencing data. HH, RS, XL, HL, JO, SR, DX, GF, and ZJ wrote the manuscript.

## Funding support

This work is the result of NIH funding, in whole or in part, and is subject to the NIH Public Access Policy. Through acceptance of this federal funding, the NIH has been given a right to make the work publicly available in PubMed Central.

- Mayo Clinic Foundation and the First Affiliated Hospital of Zhejiang University School of Medicine (to HH)
- Noncommunicable Chronic Diseases-National Science and Technology Major Project of China (2024ZD0525200, to HH and YH).
- Prostate Cancer Foundation (YIA, TO JJO).
- Department of Defense and National Cancer Institute (NCI, NIH) (K12 CA90628-23, to JJO).
- National Natural Science Foundation of China (82573200, to RS).

## Supplementary Material

Supplemental data

Unedited blot and gel images

Supplemental tables 1-4

Supporting data values

## Version 1 01/16/2026

Electronic publication
