# Supplementary material for: Reversing enhancer RNA–mediated IKBKE gene repression enables synthetic anticancer immunity in prostate cancer models
Source: J Clin Invest. 2026 Jan 16;136(2):e190928. doi: 10.1172/JCI190928 (PMC12807474; doi:10.1172/JCI190928)

Reversing enhancer RNA-mediated *IKBKE* gene repression enables synthetic anti-cancer immunity in prostate cancer models

Xiang Li, Rui Sun, Hao Li, Jacob J. Orme, Xu Zhang, Yu Hou, Sean S. Park, Yu Zhang, Yi He, Liguo Wang, Veronica Rodriguez-Bravo, Josep Domingo Domenech, Shancheng Ren, Dan Xia, Guanghou Fu, Zhankui Jia, Haojie Huang

Supplementary Figure Legends

Supplementary Figure 1. Radiation and antiandrogen dual treatment activates immune response

(**A**) Study scheme: Myc-CaP cells were injected subcutaneously into the bilateral flanks of mice. After 7 days of cell injection, tumors were treated with vehicle or ENZ (10 mg/kg) with one-side tumors treated with IR or left untreated. (**B-H**) Growth of graft tumors was measured every other day for 21 days (**B**). Tumors in each group at day 21 of cell injection were harvested and photographed (**C**) and weighted (**D**). Mouse blood was collected and PMBCs were subjected to FACS analysis (**E**, **F**). Infiltrated T and myeloid cells in tumors were isolated and analyzed by FACS. (**G**, **H**) Tumor tissues were digested and infiltrated T and myeloid cells in tumors were analyzed by FACS. Data are displayed as mean ± SD (n = 5) in **B** and **D-H**. Significance in **B** and **D-H** was determined by 2-way ANOVA.

Supplementary Figure 2. Radiation and antiandrogen dual treatment activates innate immune signaling in murine prostate cancer cells

(**A**) Heatmap showing the upregulated genes induced by dual treatment of irradiation (IR) and ENZ (10 μM) in Myc-CaP cells. (**B**) Bubble plots of the top GO terms of biological processes based on the up-regulated and down-regulated genes in (**A**). (**C**) Venn diagram showing the overlap of innate immune genes with upregulated genes by radiation and ENZ dual treatment in Myc-CaP cells. (**D**) Heatmap showing 25 upregulated genes from (**C**). (**E**, **F**) UCSC genome browser screen shot of *Isg15* and *Ifit1* gene mRNA in Myc-CaP cells treated with radiation, ENZ or combination. (**G**, **H**) qRT-PCR analysis of *Isg15* and *Ifit1* in Myc-CaP cells treated as indicated. Data are displayed as mean ± SD of triplicate experiments in **G** and **H**. Significncae in **G** and **H** was determined by one-way ANOVA, and Tukey's correction was applied for multiple comparisons.

Supplementary Figure 3. AR suppresses *IKBKE* expression via binding in an enhancer of *IKBKE* gene locus

(**A-E**) UCSC genome browser screen shot of AR ChIP-seq data at the *DDX58*, *IFI16*, *TRAF3*, *IKBKE* and *IRF5* gene loci in C4-2 cells cultured in CSS medium supplemented with vehicle or DHT (10 nM) for 24 h (GSE65066). (**F**) UCSC genome browser screen shot of RNA-seq data in the *IKBKE* gene locus in LNCaP cells cultured with CSS medium supplemented with DHT for 24 h (GSE119598). (**G**, **H**) qRT-PCR analysis of *IKBKE* mRNA expression in LNCaP cells treated with vehicle or DHT (10 nM, 24h) (**G**) or vehicle or ENZ (10 μM, 48 h) (**H**). (**I**) UCSC genome browser screen shot of AR ChIP-seq data in the *IKBKE* gene locus in LNCaP cells cultured in CSS

medium supplemented with vehicle or DHT (10 nM) for 24 h (GSE65066). (**J**) ChIP-qPCR analysis of AR occupancy at the putative *IKBKE* gene enhancer in LNCaP cells cultured in CSS medium supplemented with vehicle or DHT (10 nM) for 24 h. (**K**) Western blot analysis of expression of indicated proteins in C4-2 cells infected with lentivirus expressing control or HDAC2-specific shRNA. (**L**) 3C-qPCR analysis of chromatin looping in the IKBKE gene locus in C4-2 cells with or without HDAC2 knockdown. (**M**) ChIP-qPCR analysis of HDAC2 occupancy at the putative *IKBKE* gene enhancer in C4-2 cells treated with control or *IKBKE-e* targeting ASOs. Three biological replicates were analyzed. (**N**) UCSC genome browser screen shot of RNA-seq data for *STING1* mRNA in LNCaP and C4-2 cells treated with vehicle or ENZ (10 μM) for 1 month (GSE189966). Data are displayed as mean ± SD of triplicate experiments in **G**, **H**, **J** and **M**. Significance in **G** and **H** was determined by 2-tailed *t* test. Significance in **J** and **M** was determined by two-way ANOVA, and Tukey's correction was applied for multiple comparisons.

Supplementary Figure 4. Poly(I:C) treatment plus antiandrogen therapy activates innate immune signaling in prostate cancer cell lines

(**A**, **B**) IFC analysis of dsRNA using J2 antibody in LNCaP (**A**) and Myc-CaP cells (**B**) treated with vehicle (mock), ENZ (10 μM, 48 h), radiation or combination. (**C**, **D**) Western blot analysis of innate immune proteins in C4-2, LNCaP and Myc-CaP cell lines treated with vehicle, ENZ (10 μM) (**C**) or DHT (10 nM) (**D**) for 2 days followed by treatment with poly(I:C).

Supplementary Figure 5. Memory T cell analysis by flow cytometry in Myc-CaP tumors pre and post ENZ/IR treatment

(**A**) Representative image of graft tumors. Myc-CaP cells were injected subcutaneously into the right flank of mice. After 7 days, tumors were treated with or without IR, ENZ (10 mg/kg) and/or InVivoMAb anti-mouse PD-1 (10 mg/kg). Tumors of each group at day 21 were harvested and photographed. (**B**) Graft tumor was measured and dot plots show tumor weights in each group at day 21. (**C-F**) Memory T cells (CD4+/CD45RO+ and CD8+/CD45RO+) in PBMC before (**C**, **D**) and after indicated treatment (**E**, **F**) were analyzed by FACS. Data are displayed as mean ± SD (n = 5) in **A-F**. Significance in **A-F** was determined by 2-way ANOVA.

Supplementary Figure 6. Effect of transferring PBMC cells from treated mice into NOD SCID mice on tumor growth

(**A**, **B**) Myc-CaP cells were injected subcutaneously into the right flank of NOD SCID mice. After 7 days of cell injection, PBMC cells (5×10^6 cells/one mouse) were isolated from Myc-CaP tumor-bearing immunocompetent FVB mice treated as indicated and transferred into NOD SCID mice injected with Myc-CaP cells. Tumors of each group (n = 5) in NOD SCID at day 21 were harvested and photographed (**A**) and weighted (**B**). Data are displayed as mean ± SD (n = 5) in **B**. Significance in **B** was determined by 2-way ANOVA.

Supplementary Figure 7. Effect of CD8 and CD4 T cell depletion on growth of Myc-CaP tumors in mice

(**A**) FACS analysis of $CD4^+$ and $CD8^+$ T cells in PBMC after treated with anti-CD4 or anti-CD8 antibodies in Myc-CaP-bearing FVB mice treated as indicated. (**B**, **C**) Myc-CaP cells were injected subcutaneously into the right flank of FVB mice treated with control IgG, anti-CD4 or anti-CD8 antibodies. After 7 days, tumors were treated with IR, ENZ (10 mg/kg) and InVivoMAb anti-mouse PD-1 (10 mg/kg). Tumors in each group at day 21 were harvested and photographed (**B**) and weighted (**C**). Data are displayed as mean ± SD (n = 5) in **A** and **C**. Significance in **A** and **C** was determined by 2-way ANOVA.

Supplementary Figure 8. Effect of knockdown of Rigi or Mda5 genes on growth of Myc-CaP murine tumors in immunocompetent mice treated with ENZ/IR

(**A**, **B**) Western blot analysis of Mda5 (**A**) or Rig-i (**B**) protein in Myc-CaP cells transfected with non-specific targeting RNA (shNT) or Mda5/Rigi specific shRNAs. (**C**) Myc-CaP stable cell lines with or without knockdown of *Mda5* or *Rigi* genes were injected subcutaneously into the right flank of mice. After 7 days, tumors were treated with IR and ENZ (10 mg/kg) and/or InVivoMAb anti-mouse PD-1 (10 mg/kg). Tumors of each group at day 21 were harvested and photographed (**C**) and weighted (**D**). Data are displayed as mean ± SD (n = 5) in **D**. Significance in **D** was determined by 2-way ANOVA.

Supplementary Figure 1

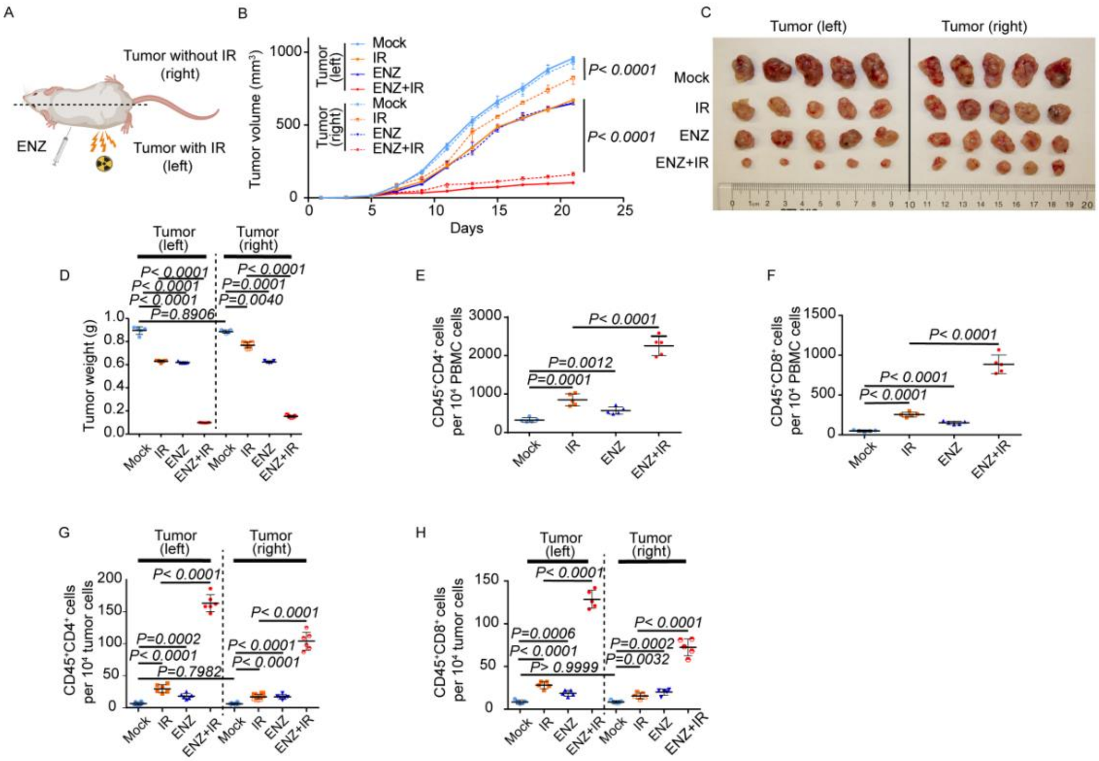

Supplementary Figure 2

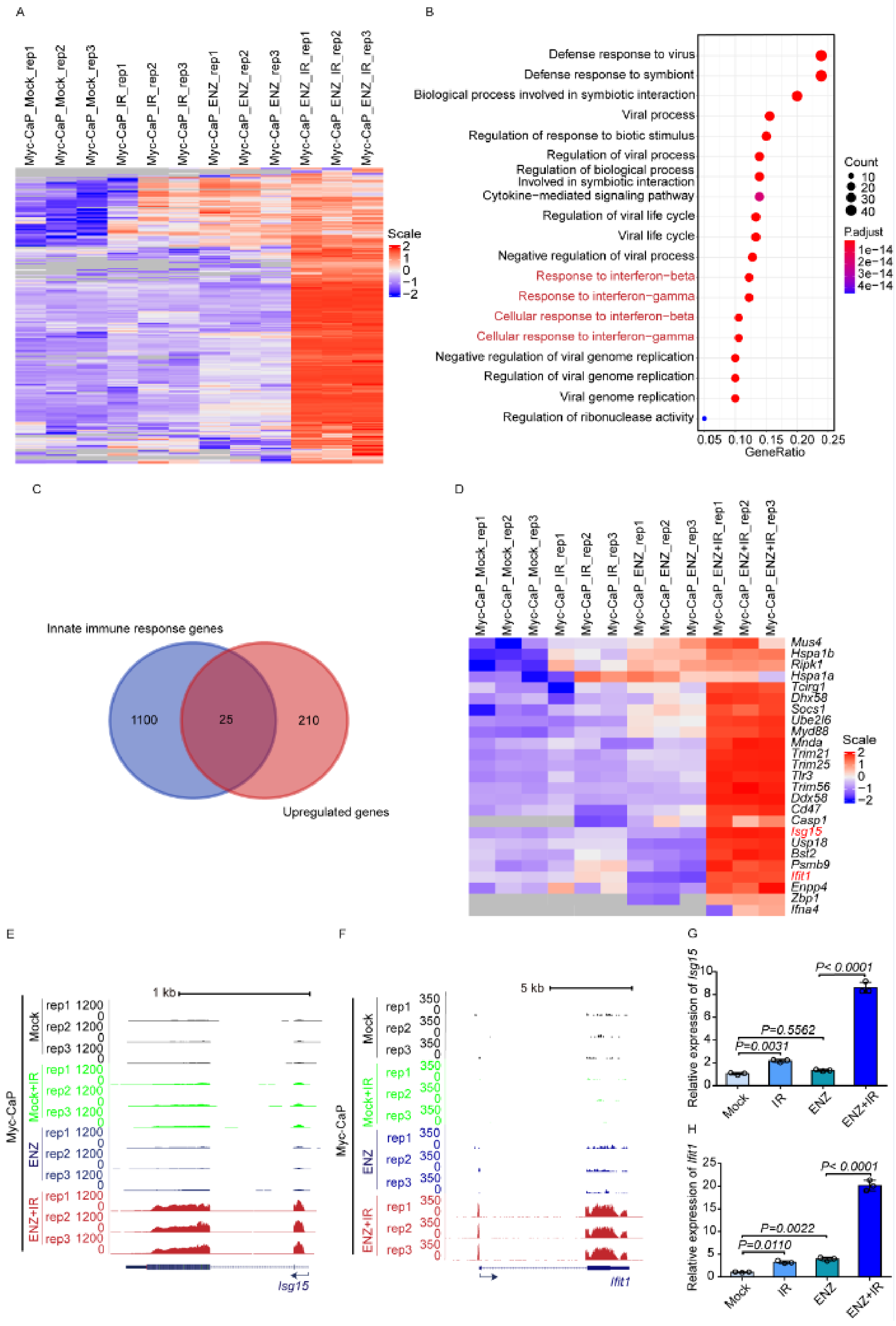

Supplementary Figure 3

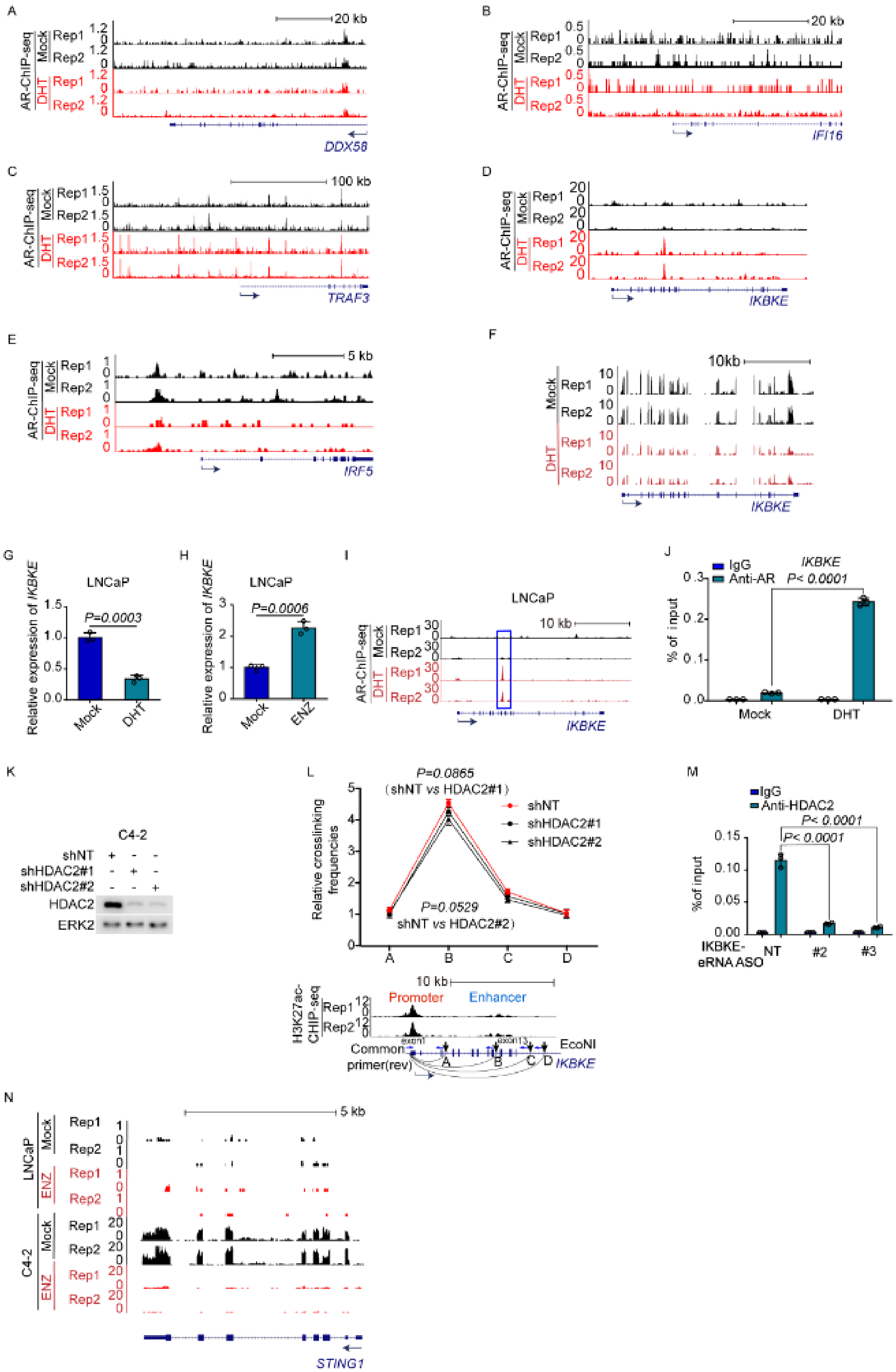

Supplementary Figure 4

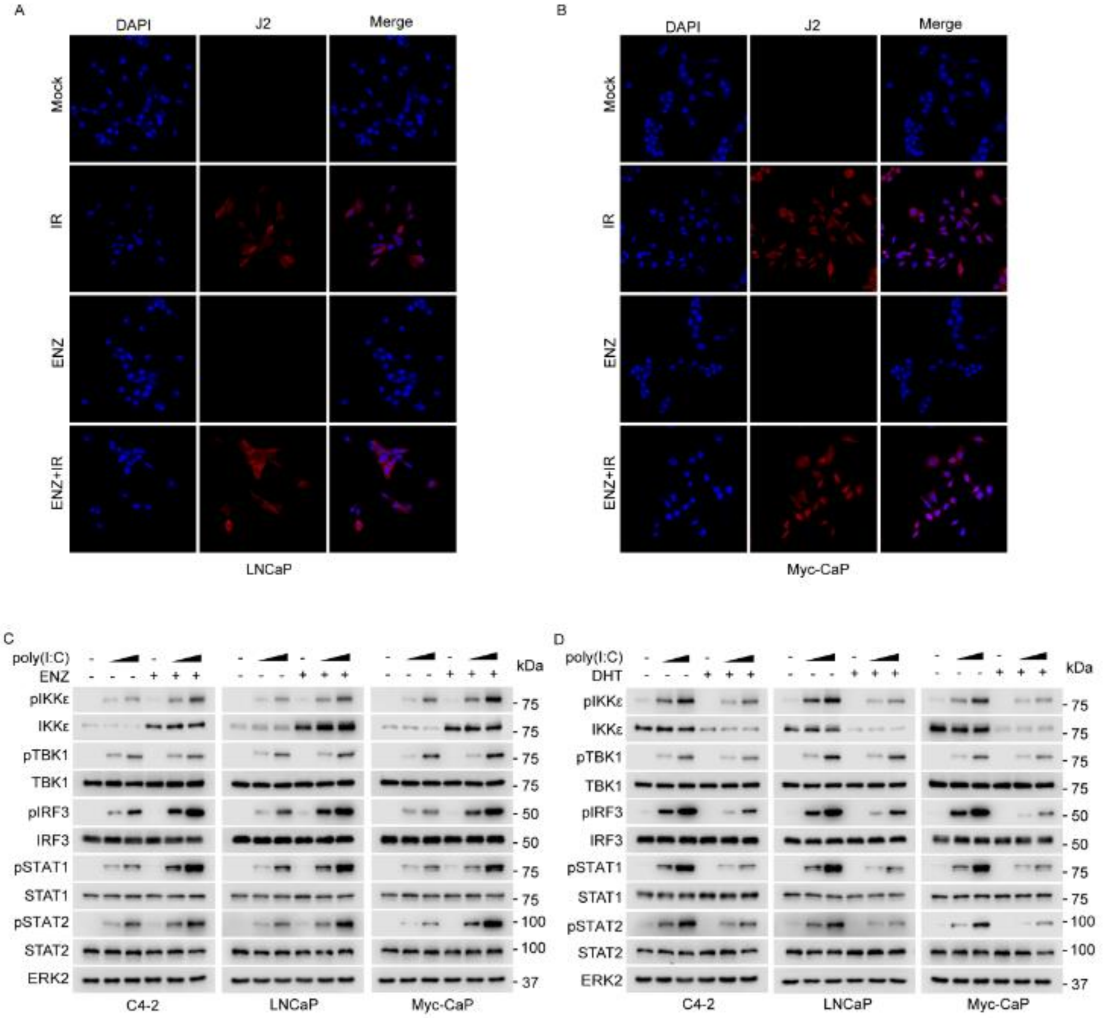

Supplementary Figure 5

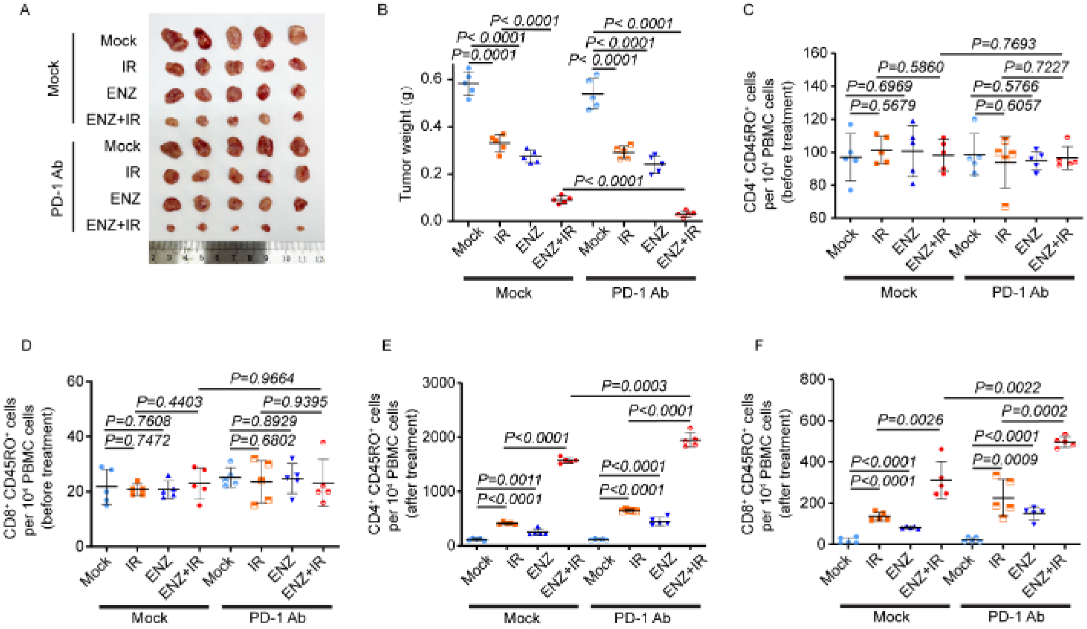

Supplementary Figure 6

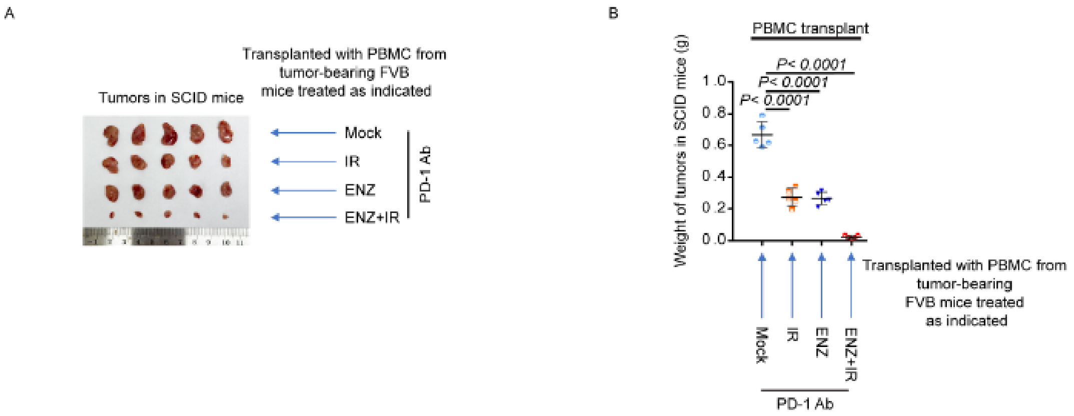

Supplementary Figure 7

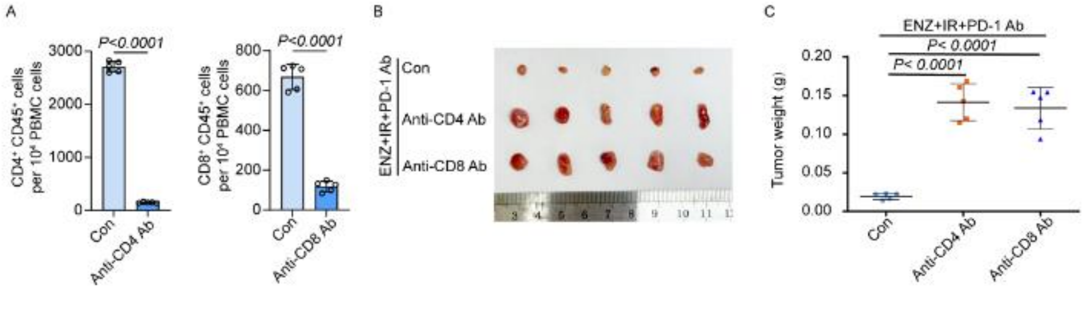

Supplementary Figure 8

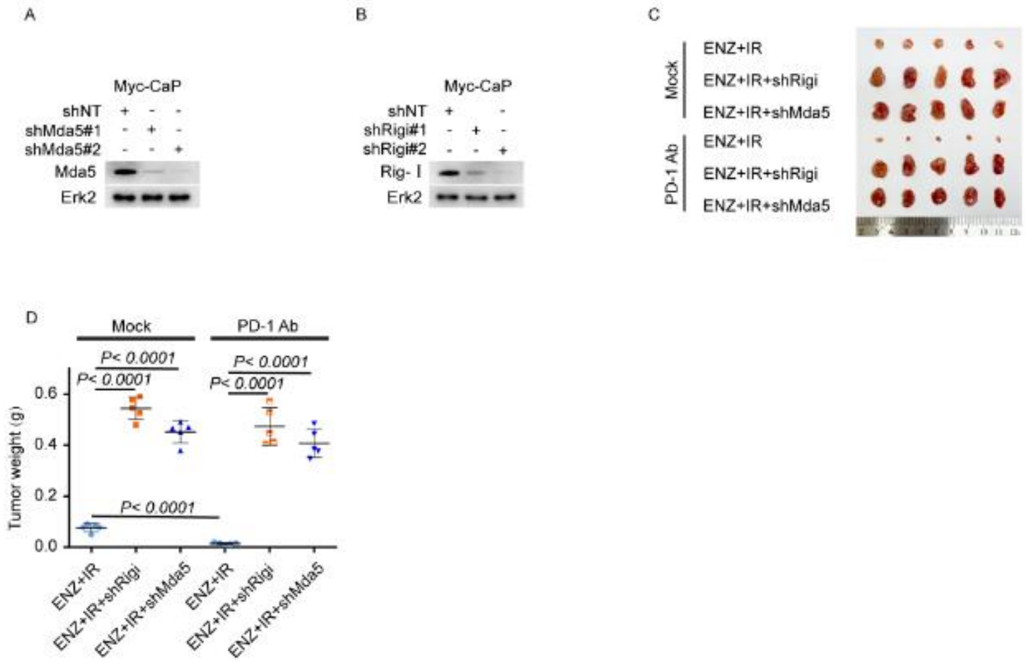

Supplement: Supplemental data [file jci-136-190928-s025.pdf]
