# Supplementary material for: Reversing enhancer RNA–mediated IKBKE gene repression enables synthetic anticancer immunity in prostate cancer models
Source: J Clin Invest. 2026 Jan 16;136(2):e190928. doi: 10.1172/JCI190928 (PMC12807474; doi:10.1172/JCI190928)

Fig. 3K

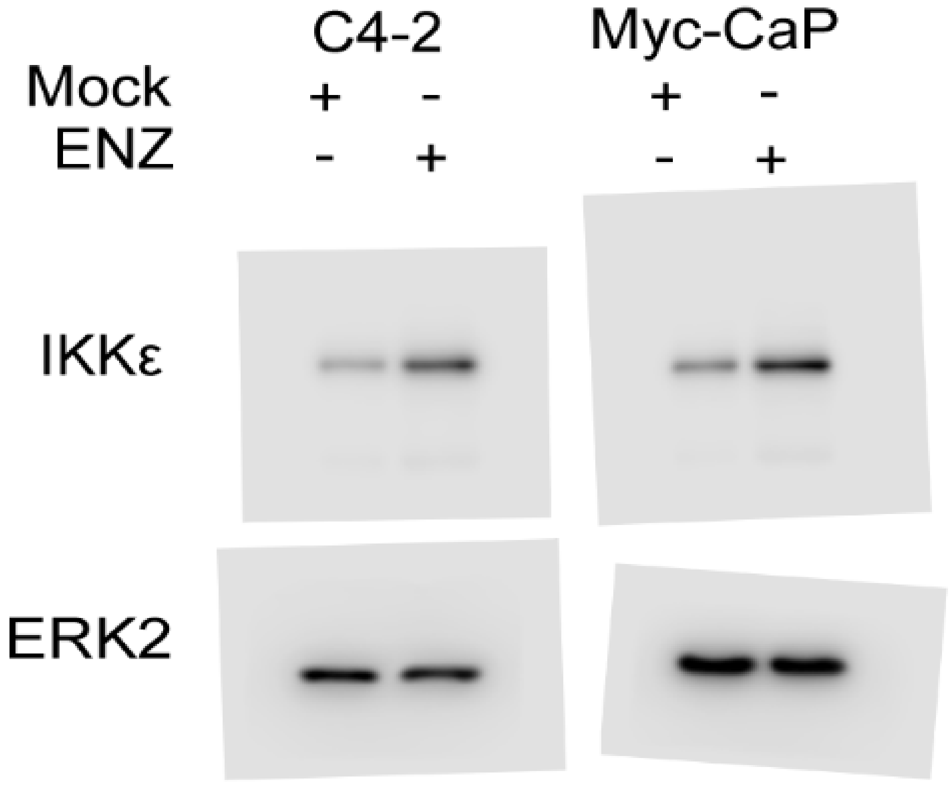

Fig. 3L

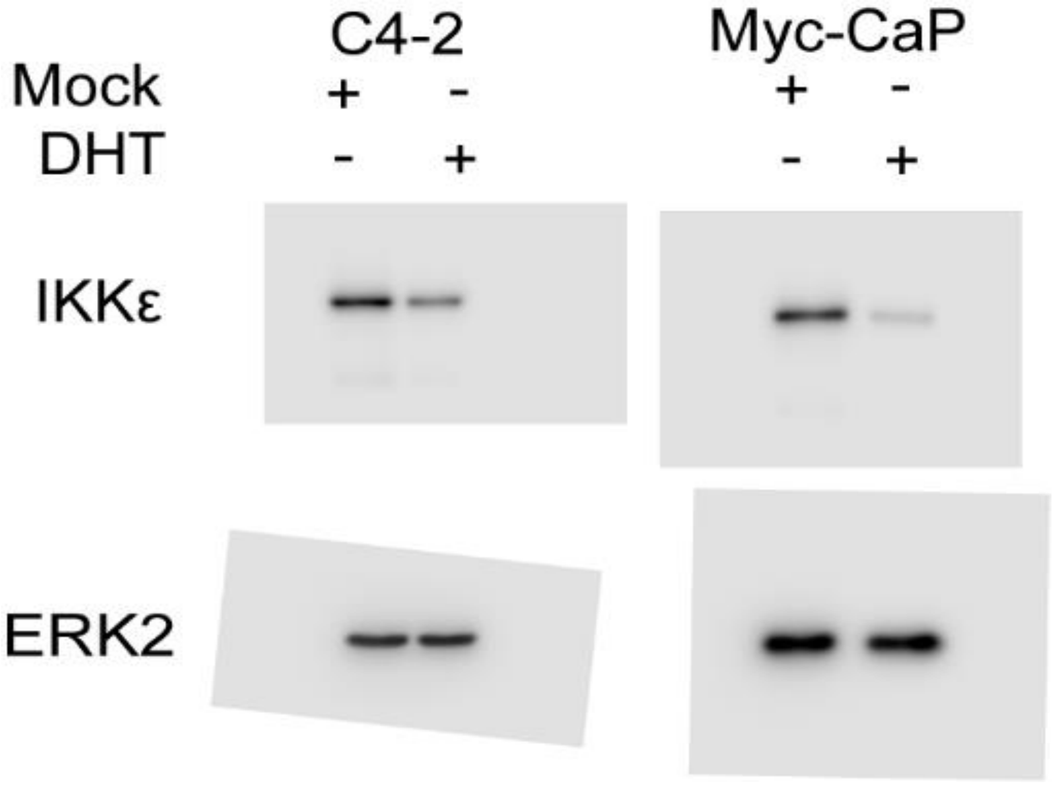

Fig. 3M

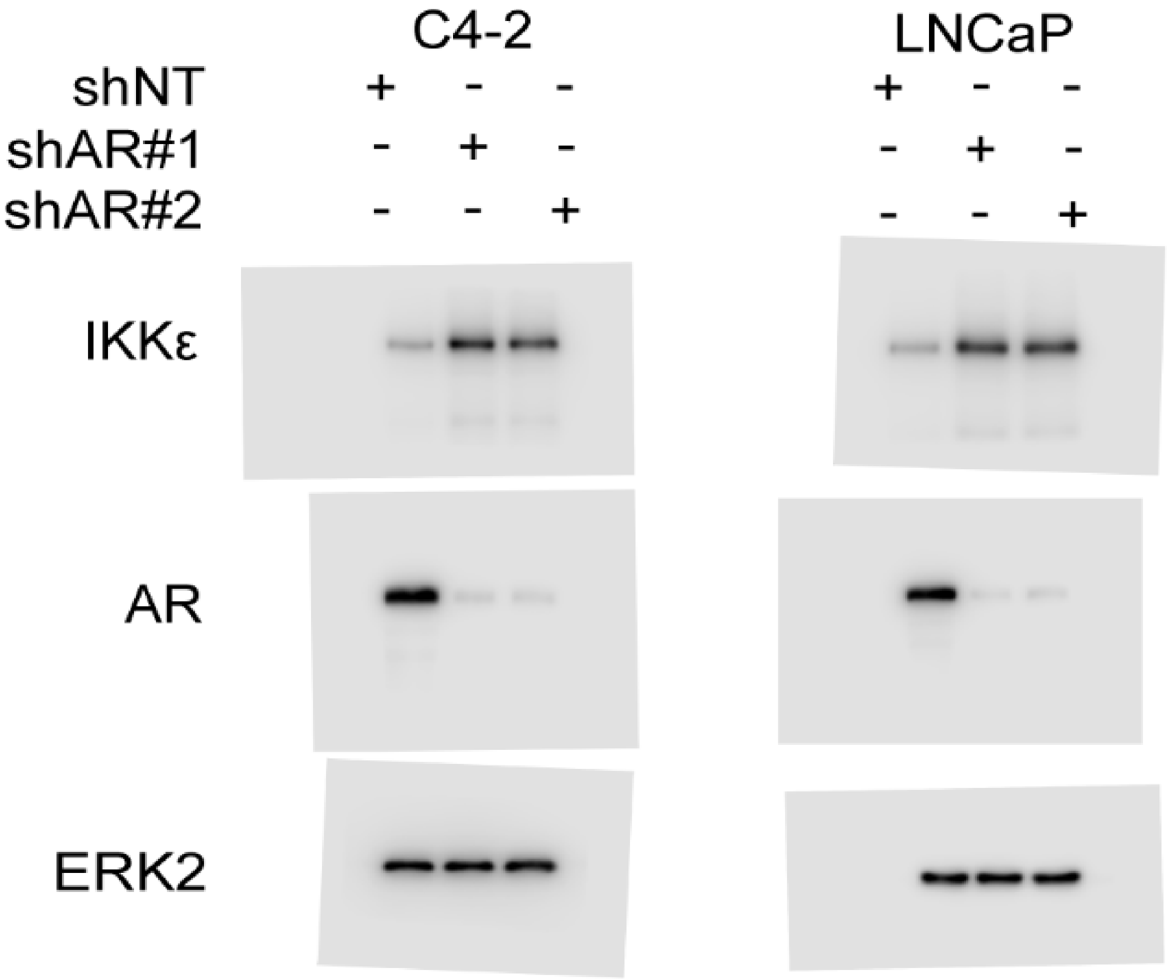
C4-2
LNCaP
shNT + - - + - -
shAR#1 - + - - + -
shAR#2 - - + - - +
IKKε
AR
ERK2

Fig. 3N

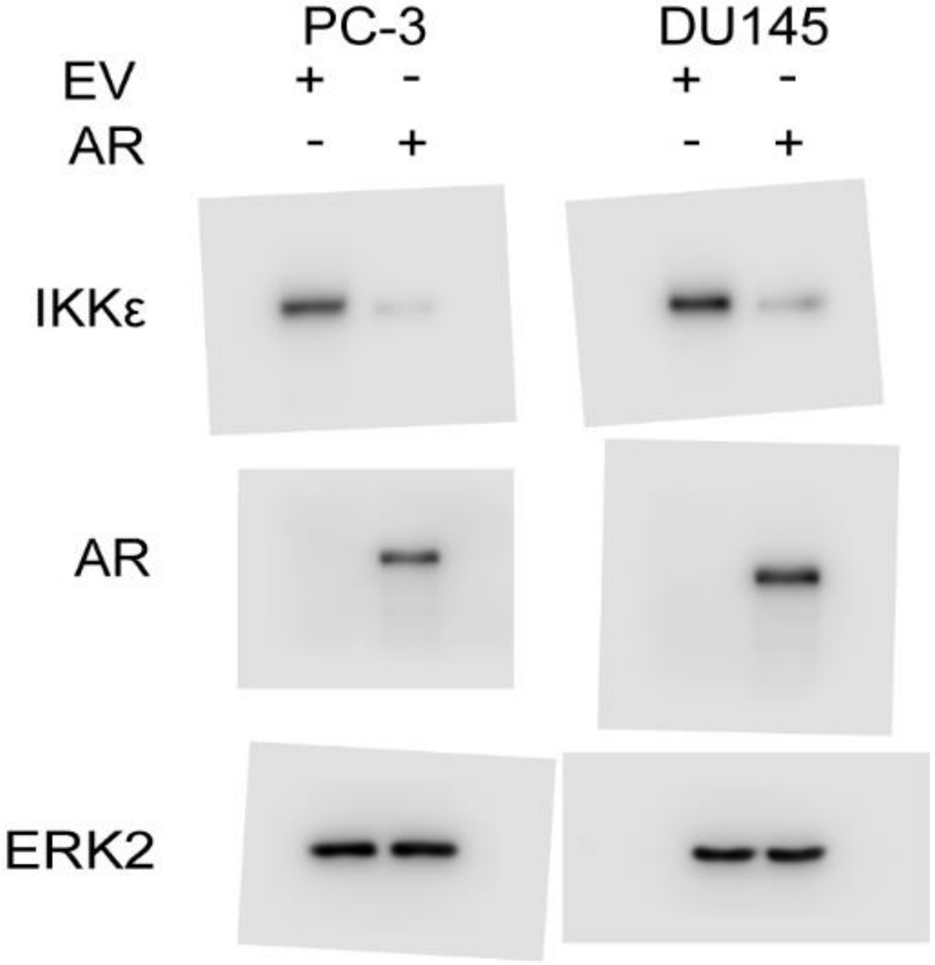
PC-3
DU145
EV + - + -
AR - + - +
IKKε
AR
ERK2

Fig. 4J

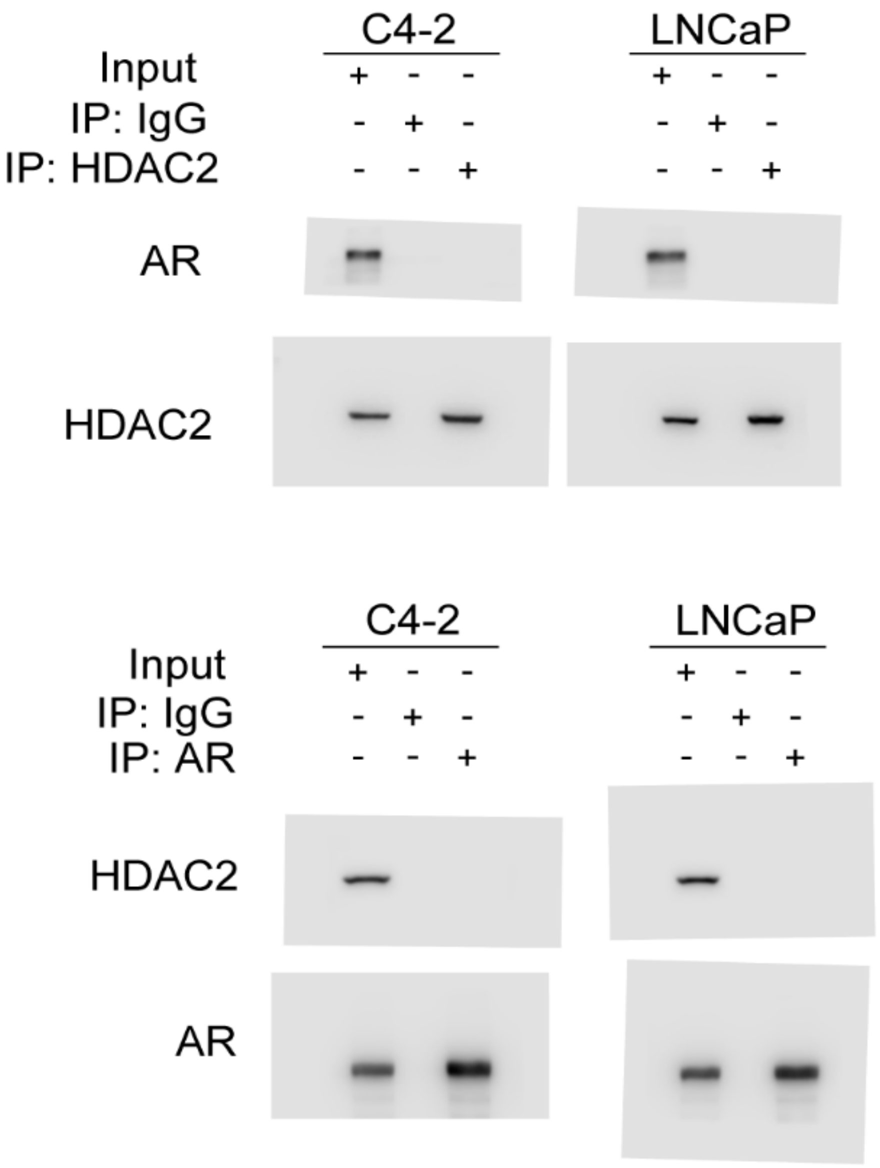
C4-2
LNCaP
Input + - - + - -
IP: IgG - + - - + -
IP: HDAC2 - - + - - +
AR
HDAC2
C4-2
LNCaP
Input + - - + - -
IP: IgG - + - - + -
IP: AR - - + - - +
HDAC2
AR

Fig. 4R

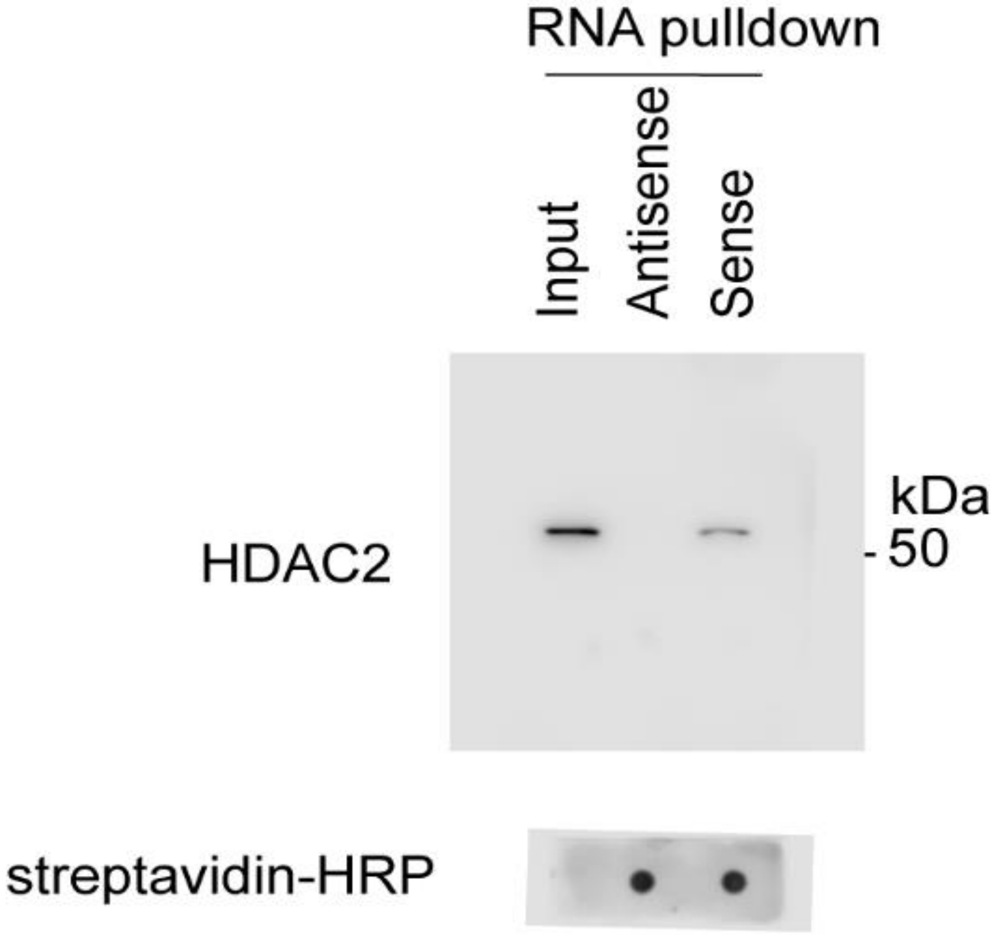
RNA pulldown
Input
Antisense
Sense
HDAC2
kDa
50
streptavidin-HRP

Fig. 4V

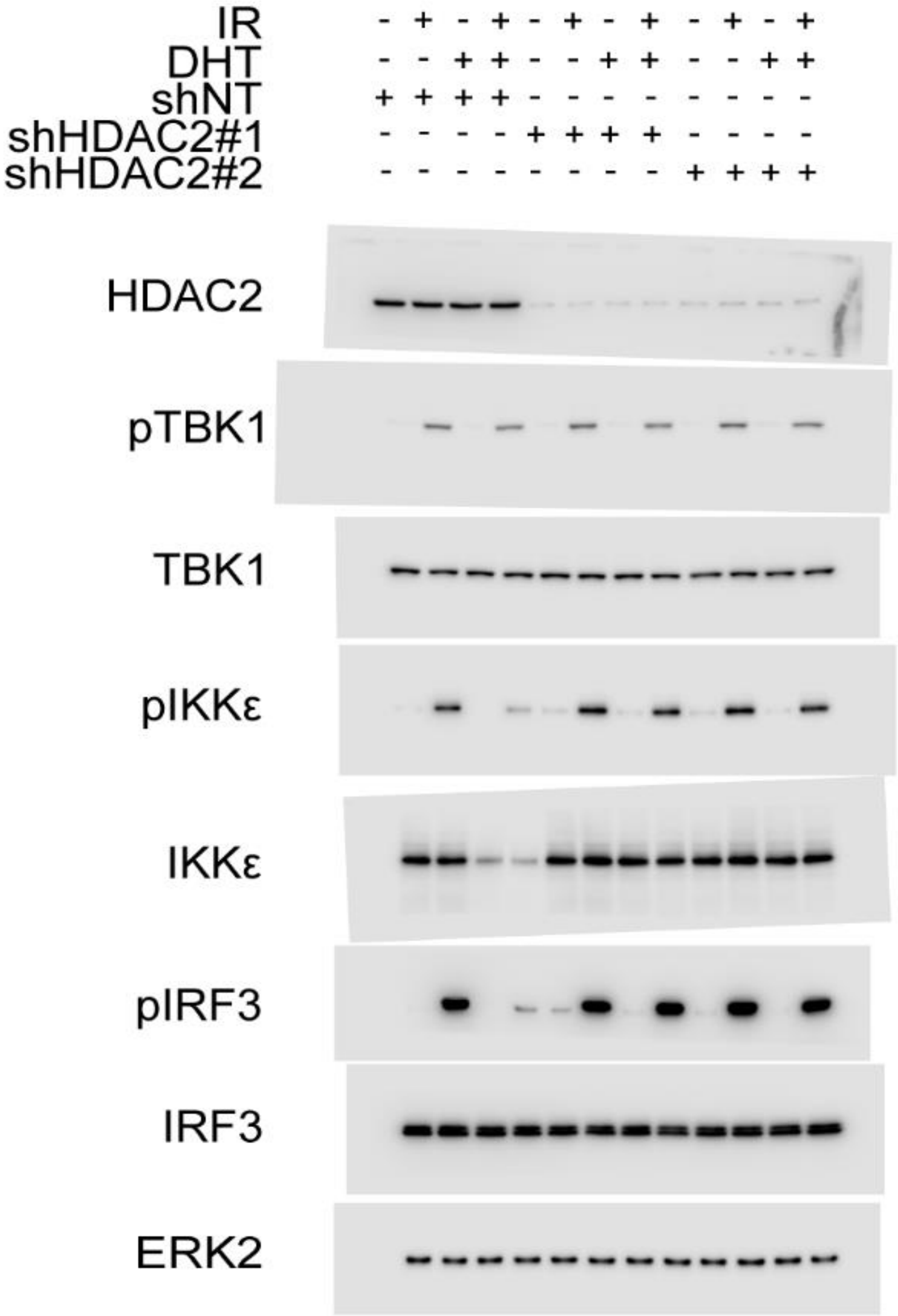
IR - + - + - + - + - + - +
DHT - - + + - - + + - - + +
shNT + + + + - - - - - - - -
shHDAC2#1 - - - - + + + + - - - -
shHDAC2#2 - - - - - - - - + + + +
HDAC2
pTBK1
TBK1
pIKKε
IKKε
pIRF3
IRF3
ERK2

Fig. 5A

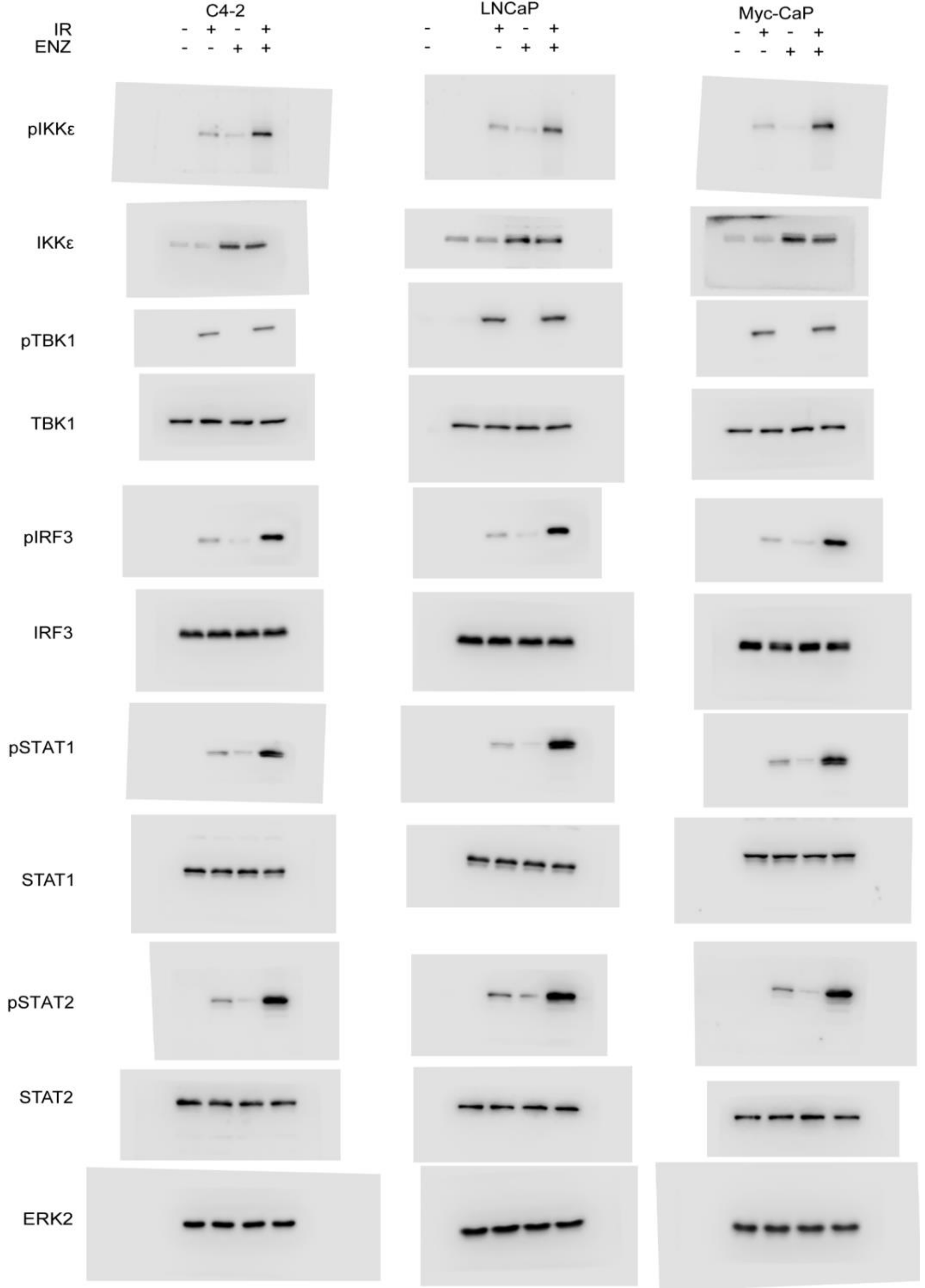

C4-2
LNCaP
Myc-CaP
IR
ENZ
pIKKε
IKKε
pTBK1
TBK1
pIRF3
IRF3
pSTAT1
STAT1
pSTAT2
STAT2
ERK2

Fig. 5B

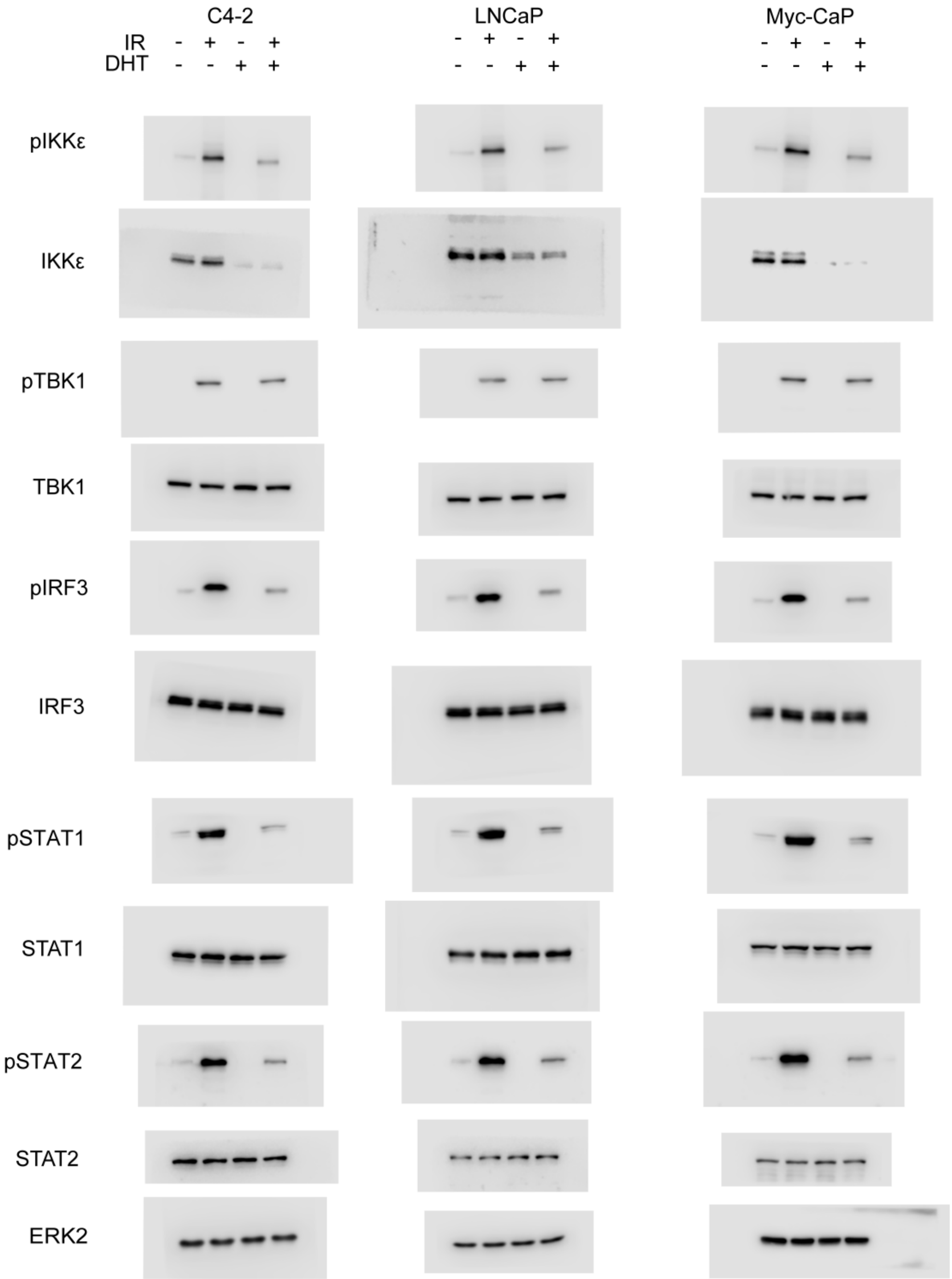
C4-2
LNCaP
Myc-CaP
IR - + - +
DHT - - + +
pIKKε
IKKε
pTBK1
TBK1
pIRF3
IRF3
pSTAT1
STAT1
pSTAT2
STAT2
ERK2

Fig. 5C

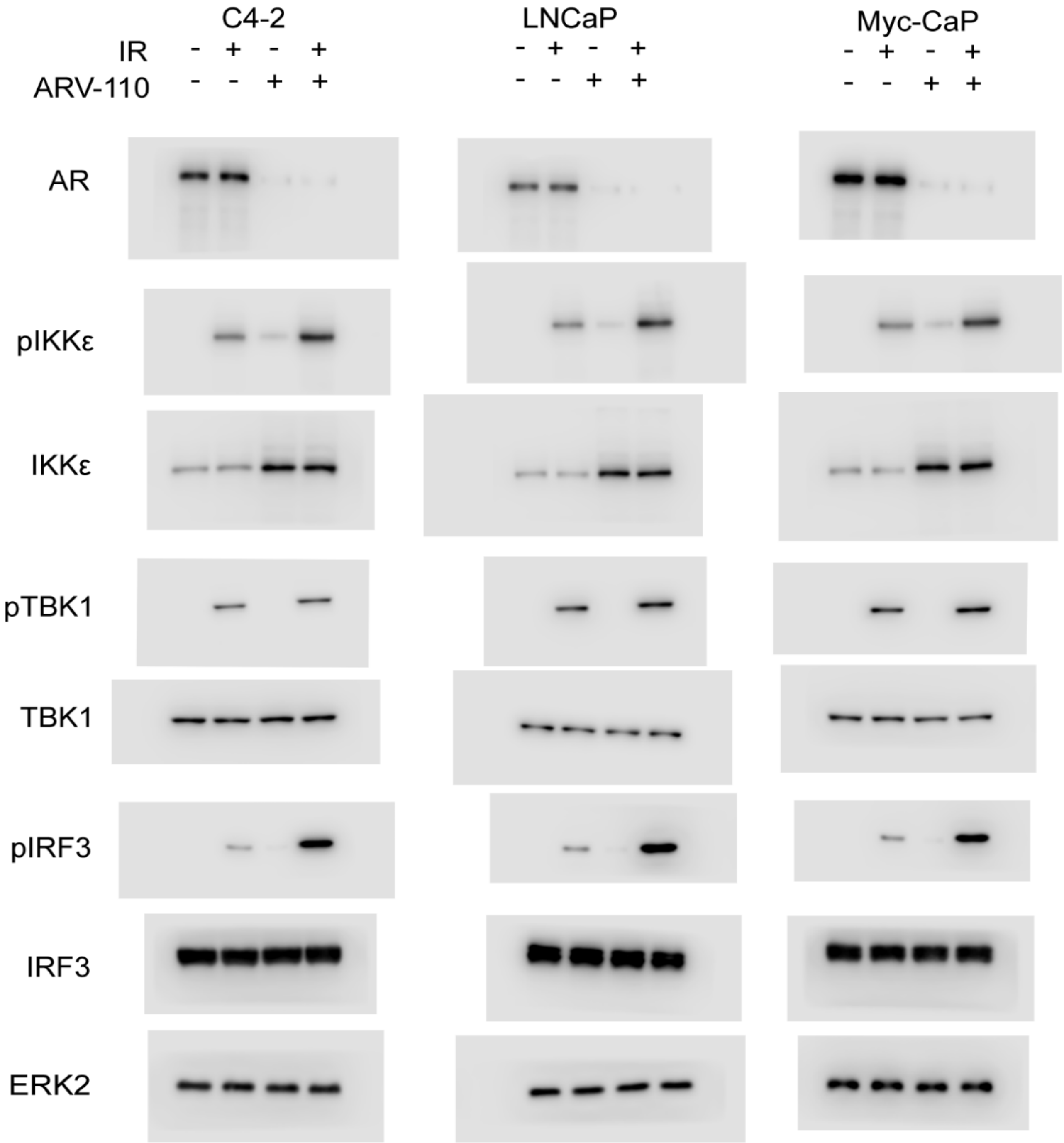
C4-2
LNCaP
Myc-CaP
IR
ARV-110
AR
pIKKε
IKKε
pTBK1
TBK1
pIRF3
IRF3
ERK2

Fig. 5H

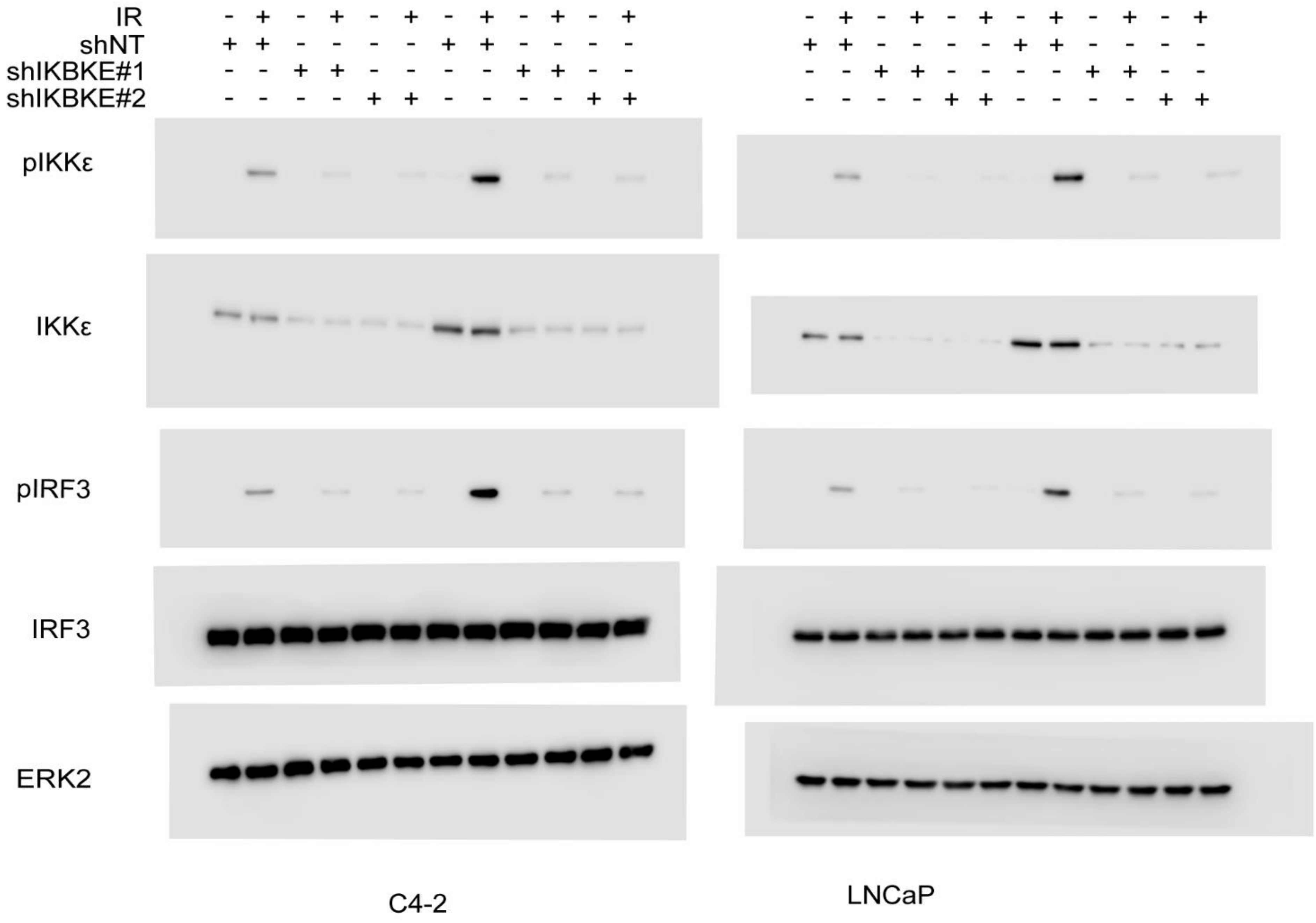

IR
shNT
shIKBKE#1
shIKBKE#2
pIKKε
IKKε
pIRF3
IRF3
ERK2
C4-2
LNCaP

Fig. 5I

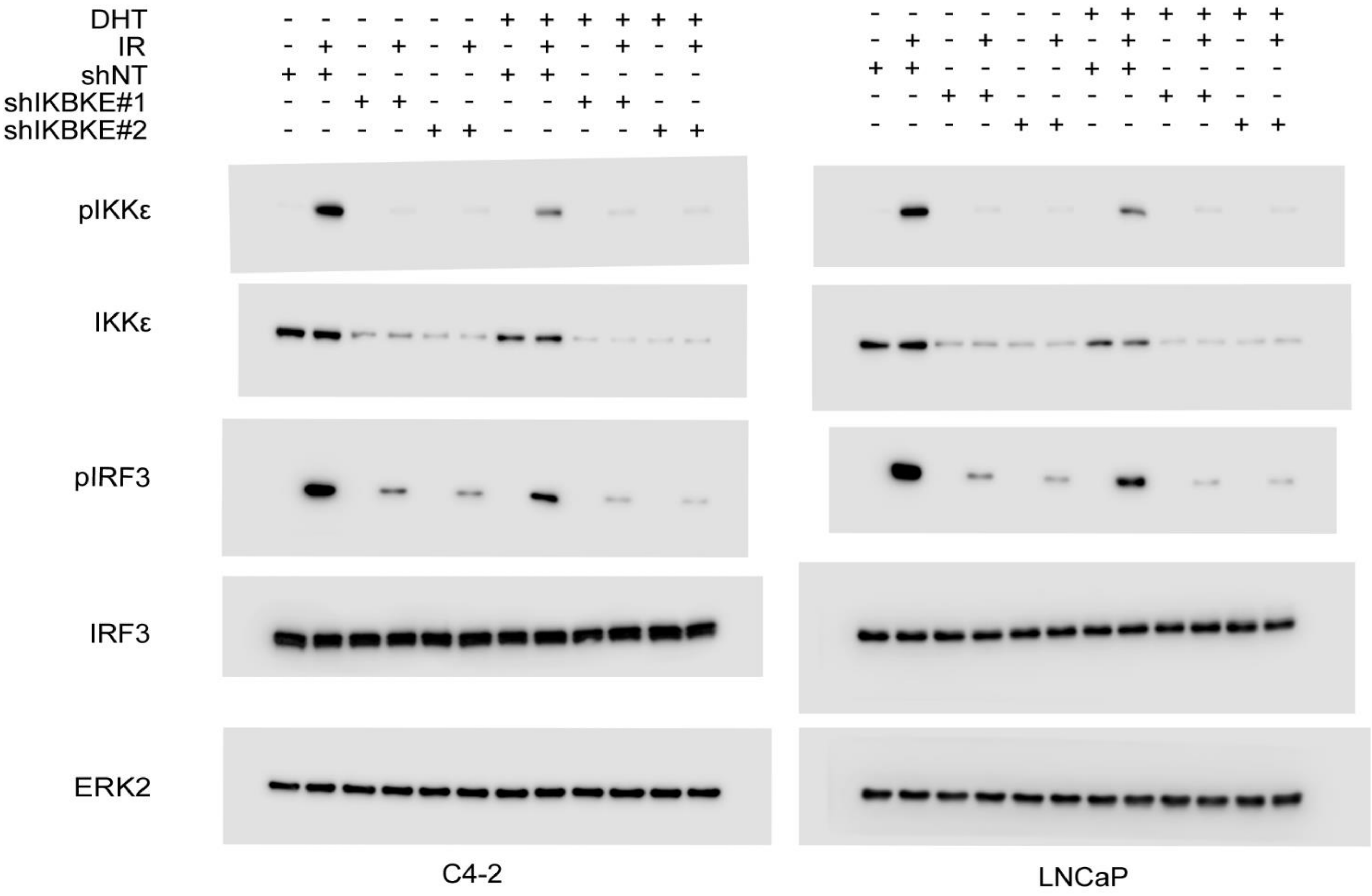
DHT
IR
shNT
shIKBKE#1
shIKBKE#2
pIKKε
IKKε
pIRF3
IRF3
ERK2
C4-2
LNCaP

Fig. 5J

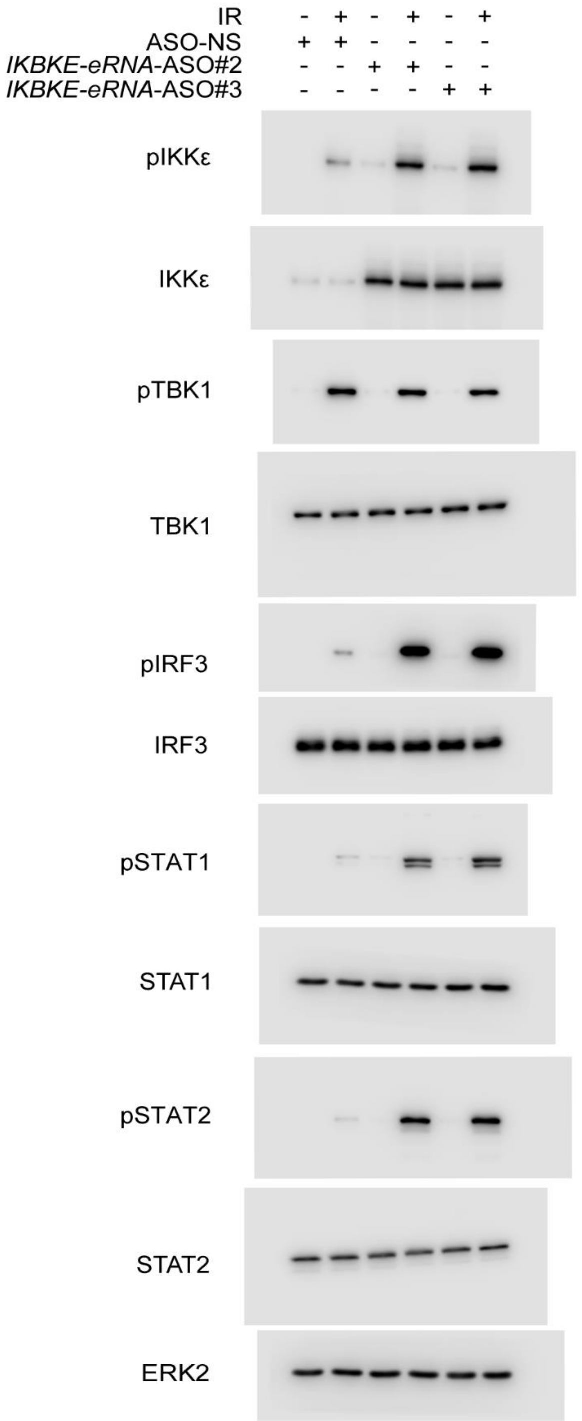
IR - + - + - +
ASO-NS + + - - - -
IKBKE-eRNA-ASO#2 - - + + - -
IKBKE-eRNA-ASO#3 - - - - + +
pIKKε
IKKε
pTBK1
TBK1
pIRF3
IRF3
pSTAT1
STAT1
pSTAT2
STAT2
ERK2

Fig. 6E

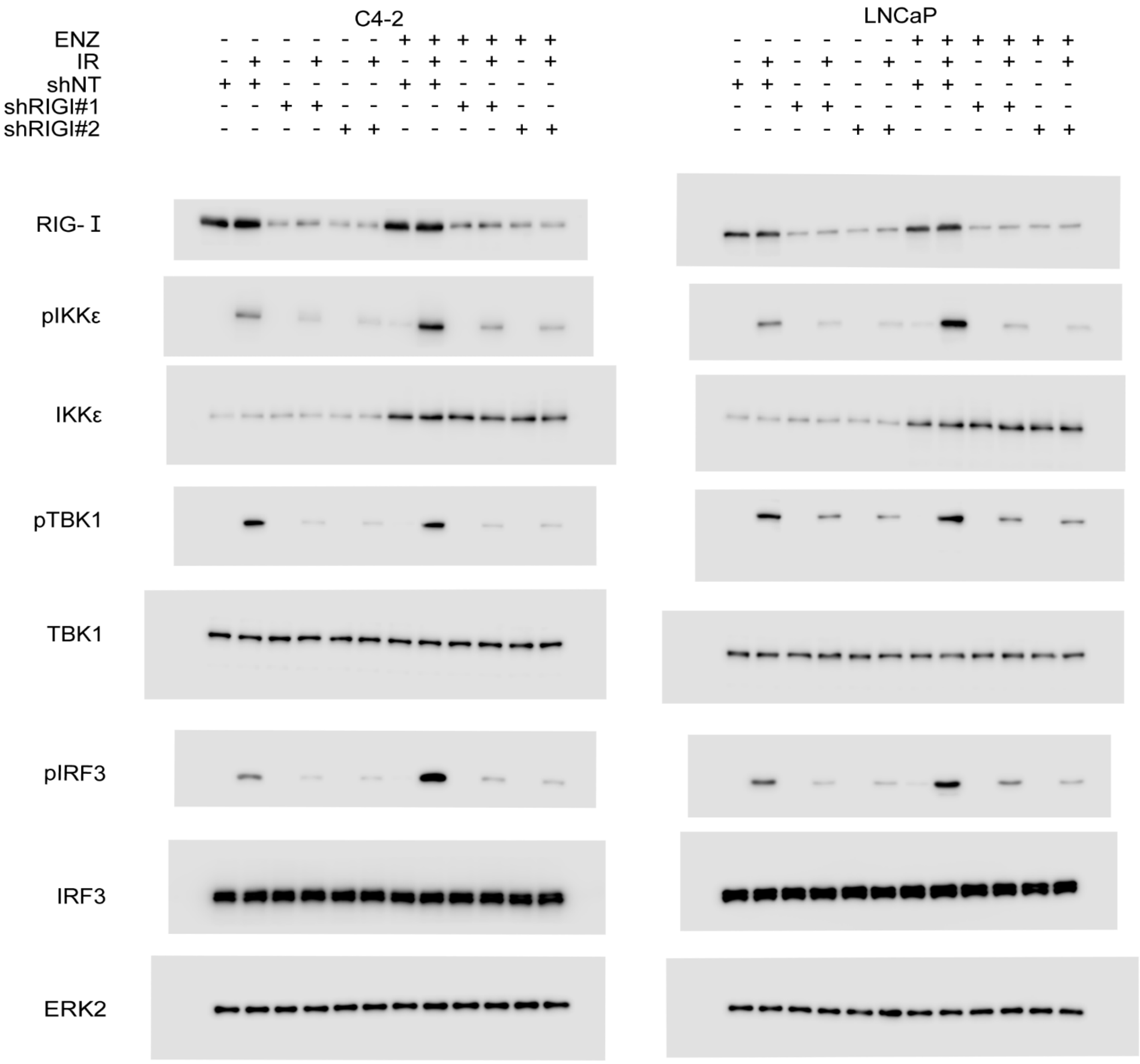
C4-2
LNCaP
ENZ
IR
shNT
shRIGI#1
shRIGI#2
RIG-Ⅰ
pIKKε
IKKε
pTBK1
TBK1
pIRF3
IRF3
ERK2

Fig. 6F

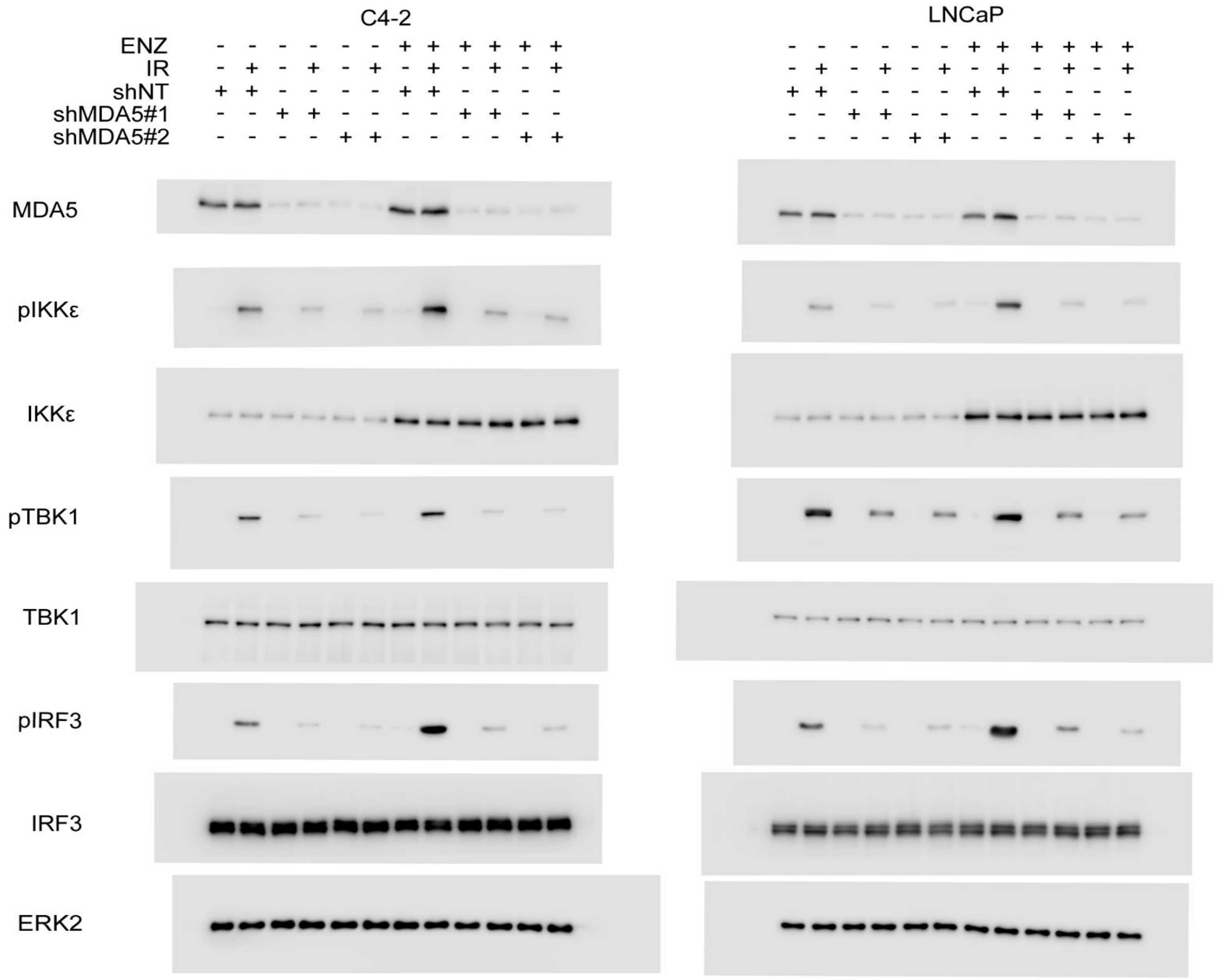
C4-2
LNCaP
ENZ
IR
shNT
shMDA5#1
shMDA5#2
MDA5
pIKKε
IKKε
pTBK1
TBK1
pIRF3
IRF3
ERK2

Fig. S3K

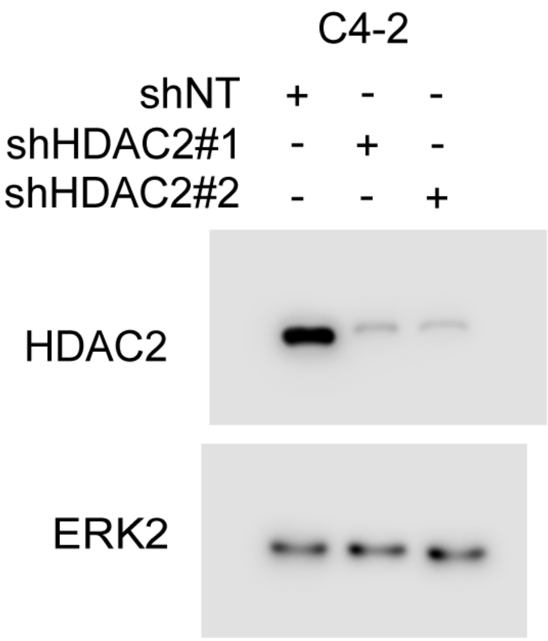

Fig. S4C

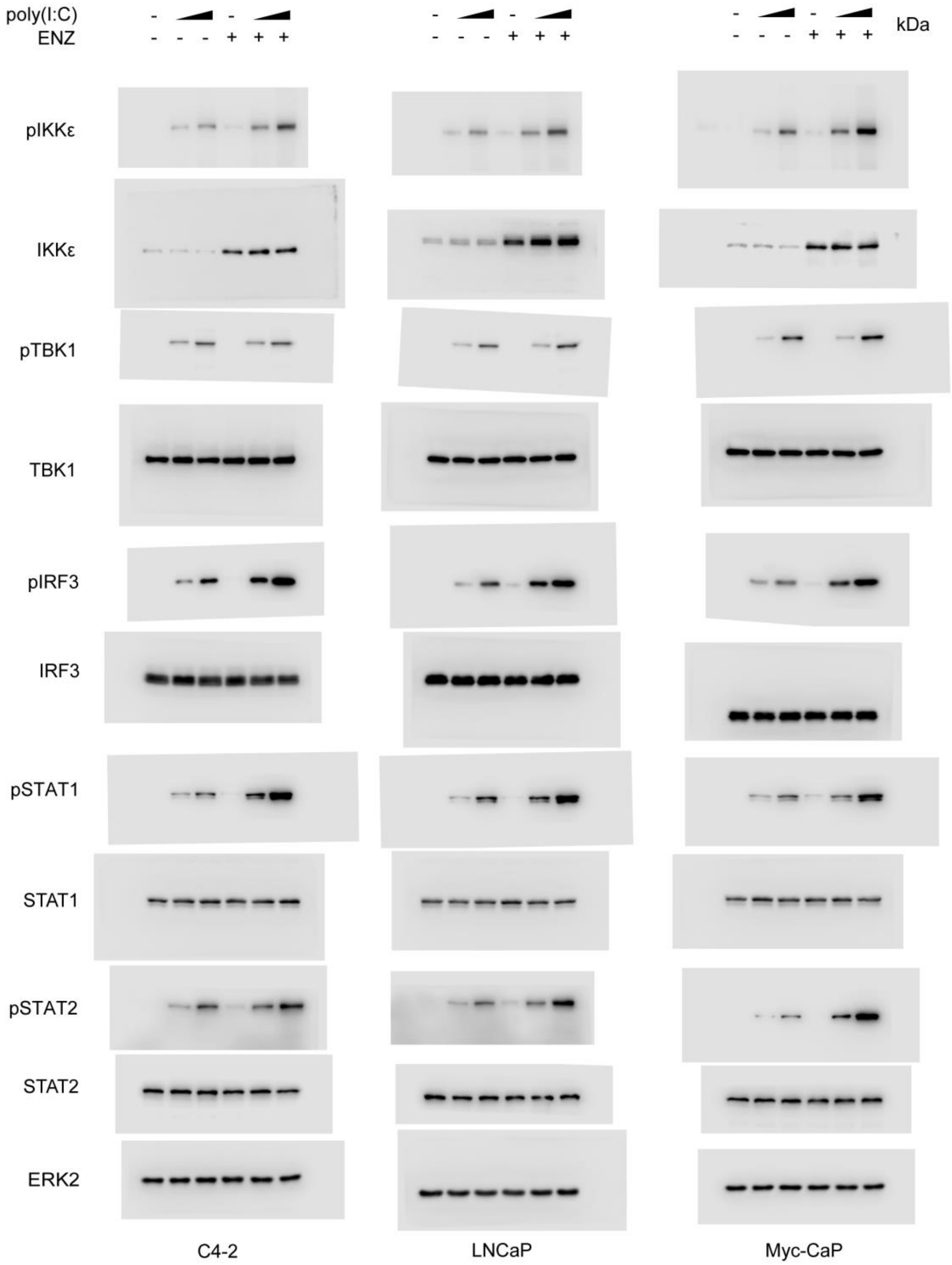

poly(I:C)
ENZ
kDa
pIKKε
IKKε
pTBK1
TBK1
pIRF3
IRF3
pSTAT1
STAT1
pSTAT2
STAT2
ERK2
C4-2
LNCaP
Myc-CaP

Fig. S4D

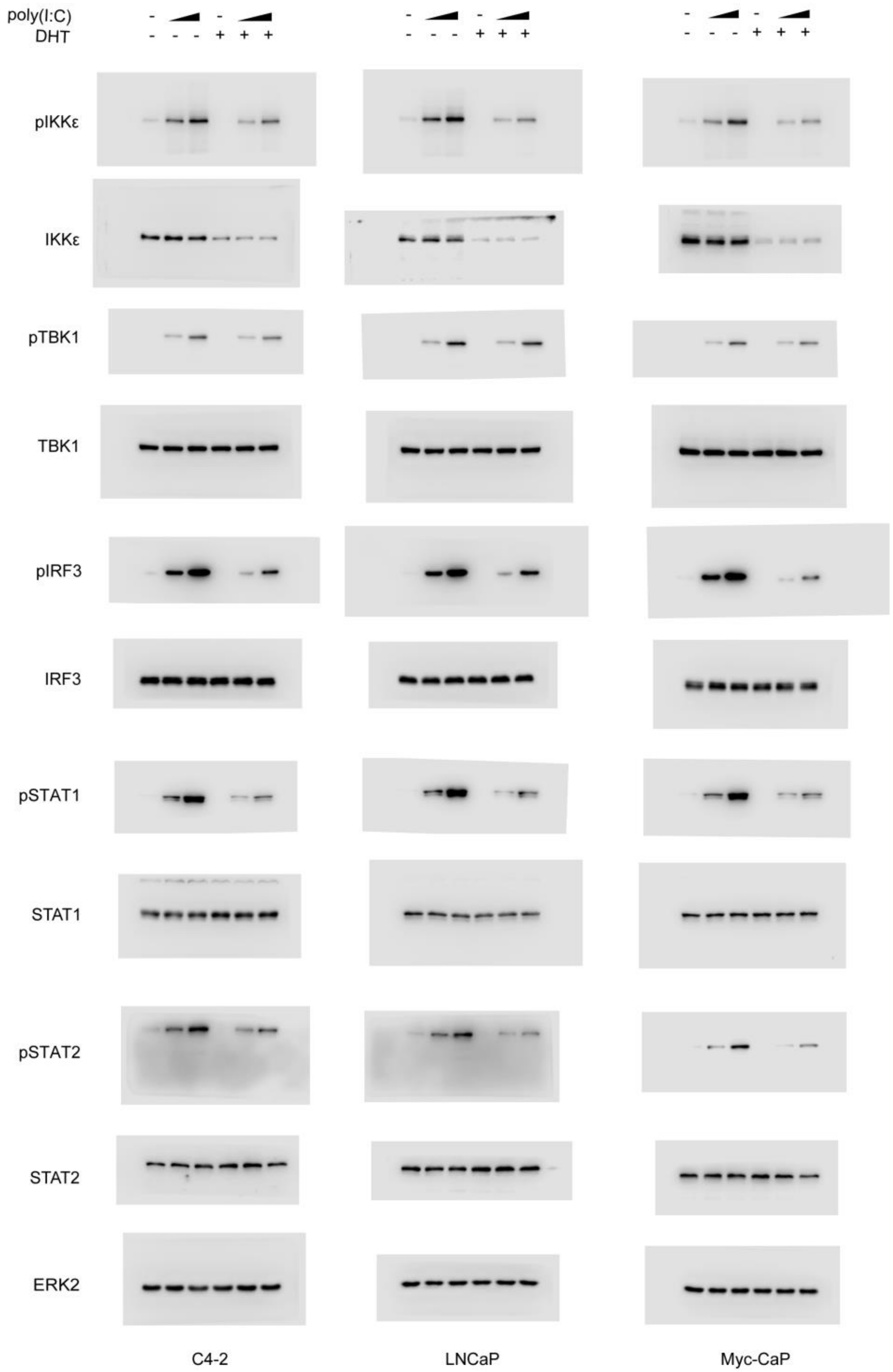
poly(I:C)
DHT
pIKKε
IKKε
pTBK1
TBK1
pIRF3
IRF3
pSTAT1
STAT1
pSTAT2
STAT2
ERK2
C4-2
LNCaP
Myc-CaP

Fig. S8A

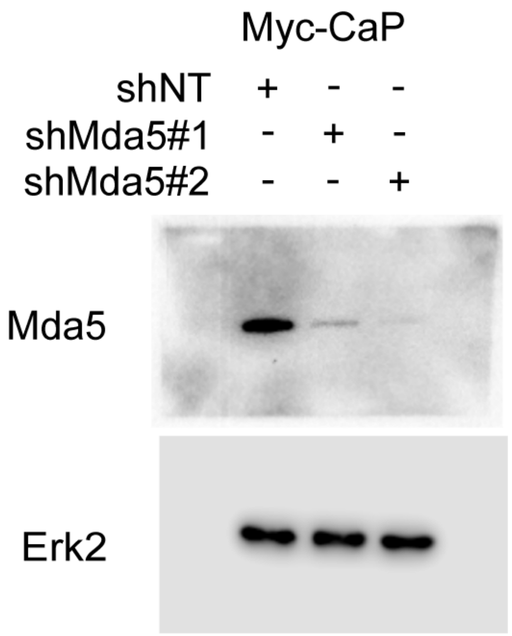

Fig. S8B

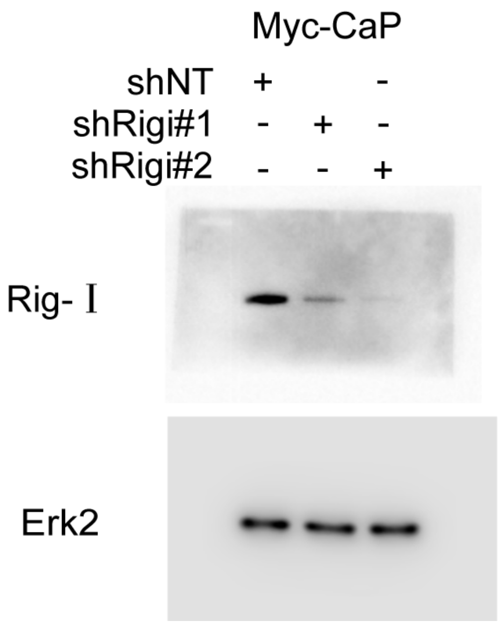

Supplement: Unedited blot and gel images [file jci-136-190928-s026.pdf]
